# Discovery of a star sensitive to the spin of Sagittarius A*

**DOI:** 10.1038/s41586-026-10894-w

**Published:** 2026-08-19

**Authors:** K. Abd El Dayem, R. Abuter, N. Aimar, P. Amaro-Seoane, A. Berdeu, J.-P. Berger, G. Bourdarot, W. Brandner, A. Burkert, D. Calderón, C. Correia, J. Cuadra, R. Davies, D. Defrère, L. Delit, A. Drescher, F. Eisenhauer, L. Esteras Otal, M. Fabricius, H. Feuchtgruber, S. Flesch, N. M. Förster Schreiber, A. Foschi, Q. Fournier, P. Garcia, R. Garcia Lopez, A. Generozov, R. Genzel, S. Gillessen, F. Gonté, X. Haubois, S. F. Hönig, M. Houllé, S. Joharle, A. Kaufer, J. Kammerer, P. Kervella, J. Kolb, L. Kreidberg, L. Labadie, S. Lacour, O. Lai, R. Laugier, J.-B. Le Bouquin, J. Leftley, R. Li, B. Lopez, D. Lutz, F. Mang, A. Mérand, F. Millour, M. Montargès, N. Morujão, H. Nowacki, M. Nowak, S. Oberti, J. Osorno, T. Ott, T. Paumard, C. Paladini, S. Pappert, H. B. Perets, K. Perraut, G. Perrin, R. Petrov, P. O. Petrucci, T. Piran, N. Pourré, S. Rabien, D. C. Ribeiro, S. Robbe-Dubois, M. Sadun Bordoni, J. Sanchez-Bermudez, D. Santos, R. Sari, J. Sauter, S. Scheithauer, J. Scigliuto, J. Shangguan, T. T. Shimizu, F. Soulez, J. Stadler, C. Straubmeier, E. Sturm, M. Subroweit, C. Sykes, L. J. Tacconi, P. Thévenet, I. Urso, F. Vincent, J. Woillez, G. Zins, GRAVITY+ Collaboration, K. Abd El Dayem, R. Abuter, N. Aimar, A. Berdeu, J.-P. Berger, G. Bourdarot, W. Brandner, C. Correia, R. Davies, D. Defrère, L. Delit, A. Drescher, F. Eisenhauer, L. Esteras Otal, M. Fabricius, H. Feuchtgruber, S. Flesch, N. M. Förster Schreiber, A. Foschi, Q. Fournier, P. Garcia, R. Garcia Lopez, R. Genzel, S. Gillessen, F. Gonté, X. Haubois, S. F. Hönig, M. Houllé, S. Joharle, A. Kaufer, J. Kammerer, P. Kervella, J. Kolb, L. Kreidberg, L. Labadie, S. Lacour, O. Lai, R. Laugier, J. Leftley, R. Li, B. Lopez, D. Lutz, F. Mang, A. Mérand, F. Millour, M. Montargès, N. Morujão, H. Nowacki, M. Nowak, S. Oberti, J. Osorno, T. Ott, T. Paumard, C. Paladini, S. Pappert, K. Perraut, G. Perrin, R. Petrov, P. O. Petrucci, N. Pourré, S. Rabien, D. C. Ribeiro, S. Robbe-Dubois, M. Sadun Bordoni, J. Sanchez-Bermudez, D. Santos, J. Sauter, S. Scheithauer, J. Scigliuto, J. Shangguan, T. T. Shimizu, F. Soulez, C. Straubmeier, E. Sturm, M. Subroweit, C. Sykes, L. J. Tacconi, P. Thévenet, I. Urso, F. Vincent, J. Woillez, G. Zins, J.-B. Le Bouquin

**Affiliations:** 1 LIRA, Observatoire de Paris, Université PSL, CNRS, Sorbonne Université Université de Paris, Meudon, France; 2 European Southern Observatory, Garching, Germany; 3 Faculdade de Engenharia, Universidade do Porto, Porto, Portugal; 4 CENTRA—Centro de Astrofísica e Gravitaçao, IST, Universidade de Lisboa, Lisbon, Portugal; 5 Universitat Politècnica de València, Valencia, Spain; 6 Max Planck Institute for Extraterrestrial Physics, Garching, Germany; 7 Université Grenoble Alpes, CNRS, IPAG, Grenoble, France; 8 Max Planck Institute for Astronomy, Heidelberg, Germany; 9 University Observatory, Faculty of Physics, Ludwig-Maximilians-Universität, Munich, Germany; 10 Max Planck Institute for Astrophysics, Garching, Germany; 11 Universidad Adolfo Ibáñez, Viña del Mar, Chile; 12 Millennium Nucleus on Transversal Research and Technology to Explore Supermassive Black Holes (TITANS), Viña del Mar, Chile; 13 Institute of Astronomy, KU Leuven, Leuven, Belgium; 14 Department of Physics, TUM School of Natural Sciences, Technical University of Munich, Garching, Germany; 15 School of Physics, University College Dublin, Dublin, Ireland; 16 Astronomy Department and Oden Institute, University of Texas at Austin, Austin, TX USA; 17 Departments of Physics and Astronomy, Le Conte Hall, University of California, Berkeley, Berkeley, CA USA; 18 European Southern Observatory, Santiago, Chile; 19 School of Physics and Astronomy, University of Southampton, Southampton, UK; 20 French-Chilean Laboratory for Astronomy, IRL 3386, CNRS and Universidad de Chile, Santiago, Chile; 21 1st Institute of Physics, University of Cologne, Cologne, Germany; 22 Université Côte d’Azur, Observatoire de la Côte d’Azur, CNRS, Laboratoire Lagrange, Nice, France; 23 Physics Department, Technion—Israel Institute of Technology, Technion—Israel Institute of Technology, Haifa, Israel; 24 Racah Institute of Physics, The Hebrew University, Jerusalem, Israel; 25 Instituto de Astronomía, Universidad Nacional Autónoma de México, Ciudad de México, México; 26 University of Lyon, Université Claude Bernard Lyon 1, ENS de Lyon, CNRS, Centre de Recherche Astrophysique de Lyon, UMR5574, Lyon, France

**Keywords:** General relativity and gravity, Compact astrophysical objects, Galaxies and clusters

## Abstract

Residing in the centre of the Milky Way, Sagittarius A* (Sgr A*) is the closest massive black hole^1^ (MBH). Its vicinity has allowed measuring individual stellar orbits around it^2–4^. The stars act as test particles and probe the gravitational potential around the 4.3 × 10^6^*M*_⊙_ MBH. These observations have determined the central mass to sub-per-cent precision^5^, and the mildly relativistic motions of stars have given access to the dominant relativistic corrections, the gravitational redshift^6,7^, the transverse Doppler effect and the prograde precession imposed by the Schwarzschild metric nature of the potential^8^. These effects are of order *β*^2^ = (*v*/*c*)^2^ (for velocity *v* and speed of light *c*). The Kerr metric for a rotating black hole leads to corrections of order *β*^3^. Here, we report the discovery of a faint main-sequence star (*m*_K_ = 19.3), S301, on an 8.7-year orbit and with small enough a pericentre distance, such that the peak velocity of the star reaches 25,000 km s^−1^. Within the measurement abilities of current near-infrared interferometry and future spectroscopy on an extremely large telescope, the motion of S301 is directly sensitive to the spin of Sgr A*. The high eccentricity of S301 suggests that it is the captured component of a binary that was torn apart by the Hills mechanism.

## Abstract (web summary)

The discovery of the fast-orbiting star S301 near Sagittarius A* offers a promising new probe of the central black hole spin of the Milky Way through relativistic orbit measurements.

## Main

Black holes in general relativity have just two additional degrees of freedom beyond mass: spin and charge. Astrophysically relevant is the spin. As all objects in the Universe rotate, we expect the same for black holes, in particular, because the angular momentum of material creating a black hole is conserved. As the effects of the spin on space–time fall off with distance *r* to the black hole at a rate of *r*^−3^, it is actually hard to measure a spin. The existence of jets in active galactic nuclei requires that the massive black holes (MBHs) located in the central engines rotate^9^. Spin estimates have been obtained from X-ray reflection spectra, in which the iron Kα line shape is a probe of the spin, albeit the impact of spin is small^10^. For accreting stellar-mass black holes, spins can be estimated from accretion theory^11^, and thus are not assumption-free. The cleanest signatures are probably those of gravitational wave mergers, in which the spins of the initial objects are among the fit parameters to the pre-mergers wave forms^12^, and the spin of the resulting black hole can be inferred from its ringdown signature^13^. The space experiment Gravity Probe B detected the spin-induced precession because of Earth twisting space–time with 5*σ* significance^14^, testing the far-field and slow-motion approximation around a massive body, which equals the approximation of the Kerr metric in the same limit^15^. Overall, there are only few observational constraints on the spin parameter of the Kerr metric.

Sagittarius A* (Sgr A*), the closest MBH in the Galactic Center (GC) at a distance of 8.3 kpc offers a direct, dynamical way to measure its spin. The observation of stellar orbits has made Sgr A* one of the best cases for the existence of black holes in general. A few dozen stars revolve on (nearly) Keplerian orbits, with an almost relaxed eccentricity distribution and randomly oriented orbits. Most valuable are the stars that come closest to Sgr A*, as they probe deepest into the gravitational potential. In particular, the star S2 on a 16-year orbit^2^ has been in focus, because of its comparably easily accessible orbit. The motion of S2 is notably affected by relativistic effects: During its 2018 pericentre passage, the gravitational redshift of Sgr A* led to an additional change of measured atomic line positions of about 200 km s^−1^ (refs. ^6,7^). By 2020, the astrometric data of the star showed that the orbit had precessed in 2018 by around 12′, fully consistent with a motion in the Schwarzschild metric^8^. Key for these discoveries was the advent of near-infrared interferometry with the GRAVITY instrument at the Very Large Telescope of the European Southern Observatory^16^. At the *β*^3^ order, the leading-order term from the Kerr metric contributes, and the motion is sensitive to the spin of Sgr A*, often called Lense–Thirring precession^17–20^.

For S2, current instrumentation requires prohibitively long time series to detect the spin. The parameter determining how sensitive a star is to relativistic effects is the pericentre distance *r*_p_ = *a*(1 − *e*) (ref. ^21^), which for S2 is 1,400 *R*_S_ (Schwarzschild radii, 1*R*_S_ = 10 μas for Sgr A*). Stars with smaller *r*_p_ would allow a quicker detection, requiring smaller semi-major axes *a* and/or larger eccentricities *e*. Given a spatial resolution of about 1.7 mas, an astrometric precision of about 30 μas comparable to *R*_S_ and a field of view of approximately 70 mas, GRAVITY can trace stars with pericentre passages much closer to Sgr A* than S2. The newly discovered star S301 constitutes an example for such a relativistic test particle suitable for measuring the spin of Sgr A*.

## Observations and data analysis

**Extended Data Fig. 1:**
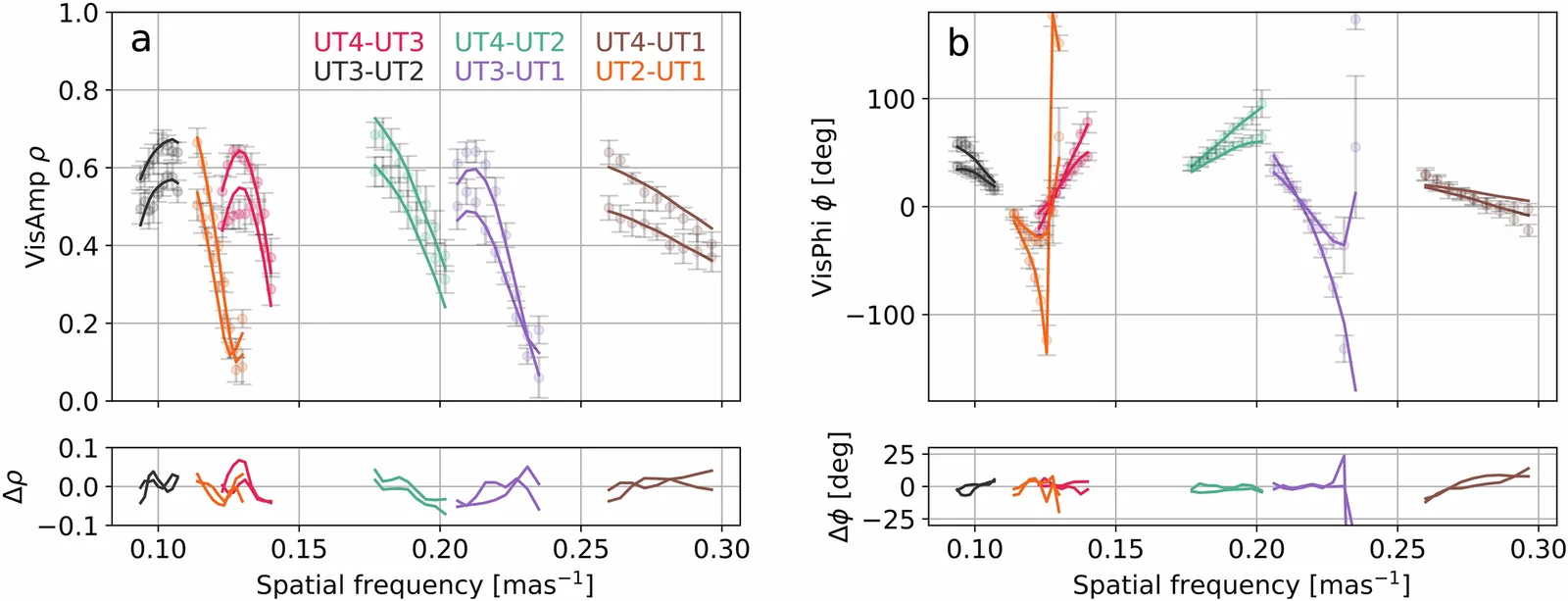
Reduced GRAVITY data consisting of complex visibilities. GRAVITY GC data, showing the complex visibility samples of a single GRAVITY exposure from 2023-07-04 with 360 s integration time, represented as amplitude *ρ* and absolute phase *ϕ*, plotted against spatial frequency. The color scheme encodes the corresponding baseline of the VLTI for two linear polarizations, each with nine spectral channels. The solid lines depict the model as reconstructed from a total of 16 exposures.

Since 2017, we have regularly observed the central arcsecond around Sgr A* with GRAVITY to track the stellar motions. Observations take place monthly during roughly week-long campaigns between March and September (Extended Data Table 1). A total of around 80–100 h yr^−1^ of observing time is used for that. Most of it is spent on a central pointing containing Sgr A*, as this serves as astrometric reference for all stellar positions. The data consist per 360 s exposure of a set of complex visibilities for six baselines, sampled at 12 spectral channels for the two linear polarizations (Extended Data Fig. 1).

**Fig. 1:**
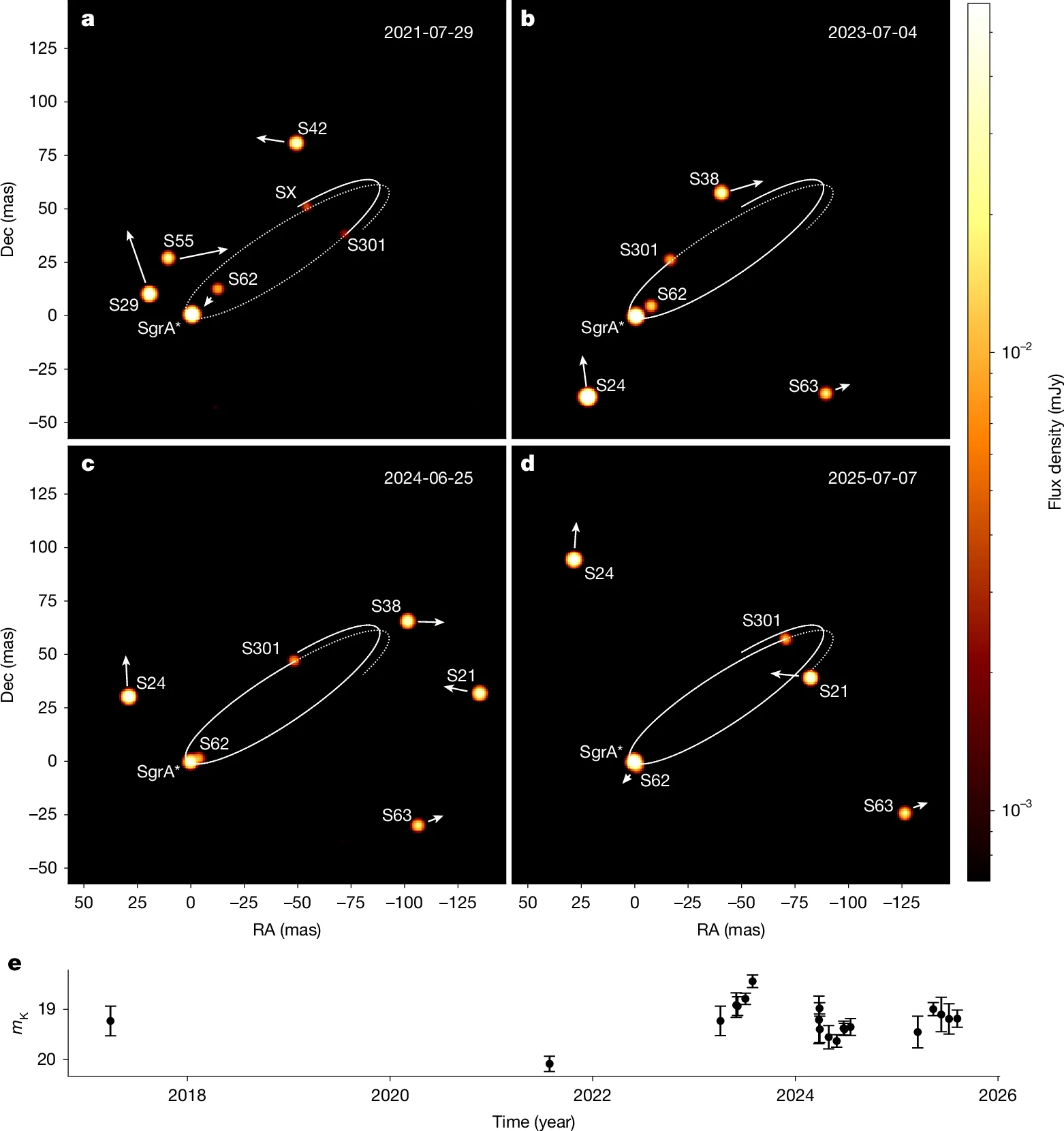
GRAVITY images of the region around Sgr A*. **a**–**d**, Annotated time series from 2021 to 2025, reconstructed with G^R^. For S301, the orbital trace (Fig. 2) is shown on top. In **c** and **d**, Sgr A* and S62 are confused in this representation because of the smoothing of the image with a Gaussian kernel. SX in the 2021 image is a potential source not yet rediscovered. **e**, K-band light curve of S301.

**Extended Data Fig. 2:**
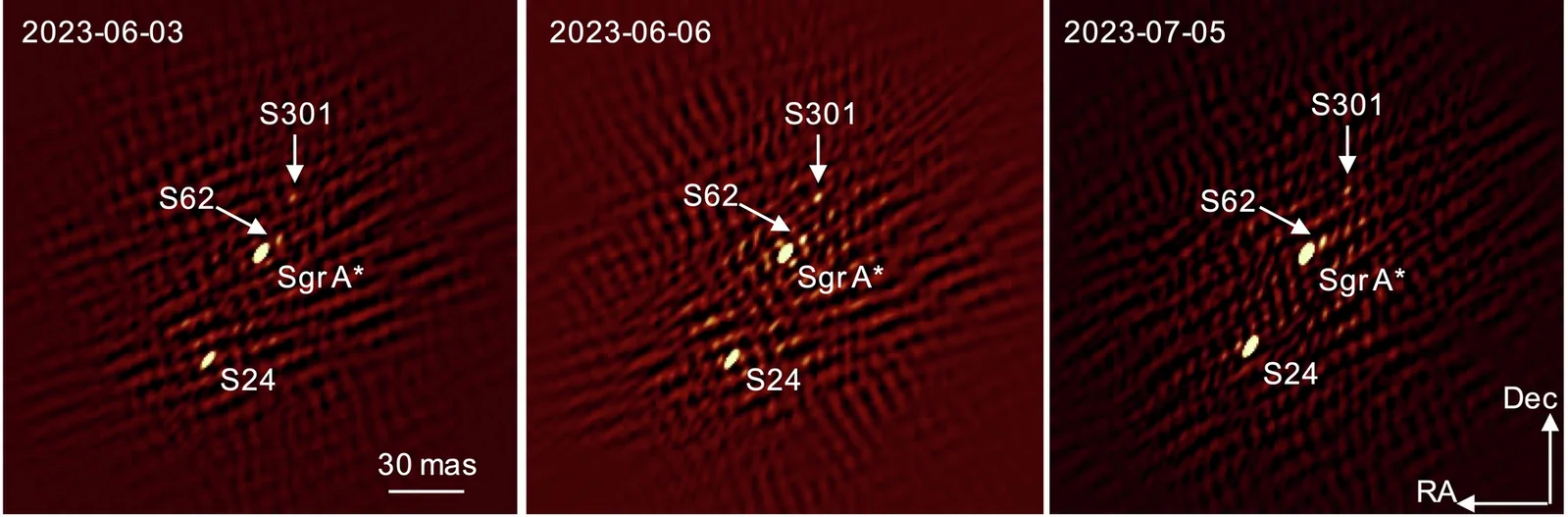
S301 detections using the CLEAN algorithm. Three examples of inferring S301 in 2023 using the CLEAN algorithm implemented in AIPS^70^, following^51^ and^22^.

We group the data per night and analyse the datasets in two ways: First, we fit a model to them. It contains the known sources and returns positions and fluxes of these. By design, this does not reveal new sources. Second, we used image reconstruction to perform Fourier-inversion of the data into an image. Beyond classical algorithms developed for radio interferometry such as CLEAN (for an example, see Extended Data Fig. 2), we use our own code ‘GRAVITY-RESOLVE’, G^R^ (ref. ^22,23^; F. Mang et al., manuscript in preparation; Methods). The images allow identifying previously unknown sources (Fig. 1).

**Extended Data Fig. 3:**
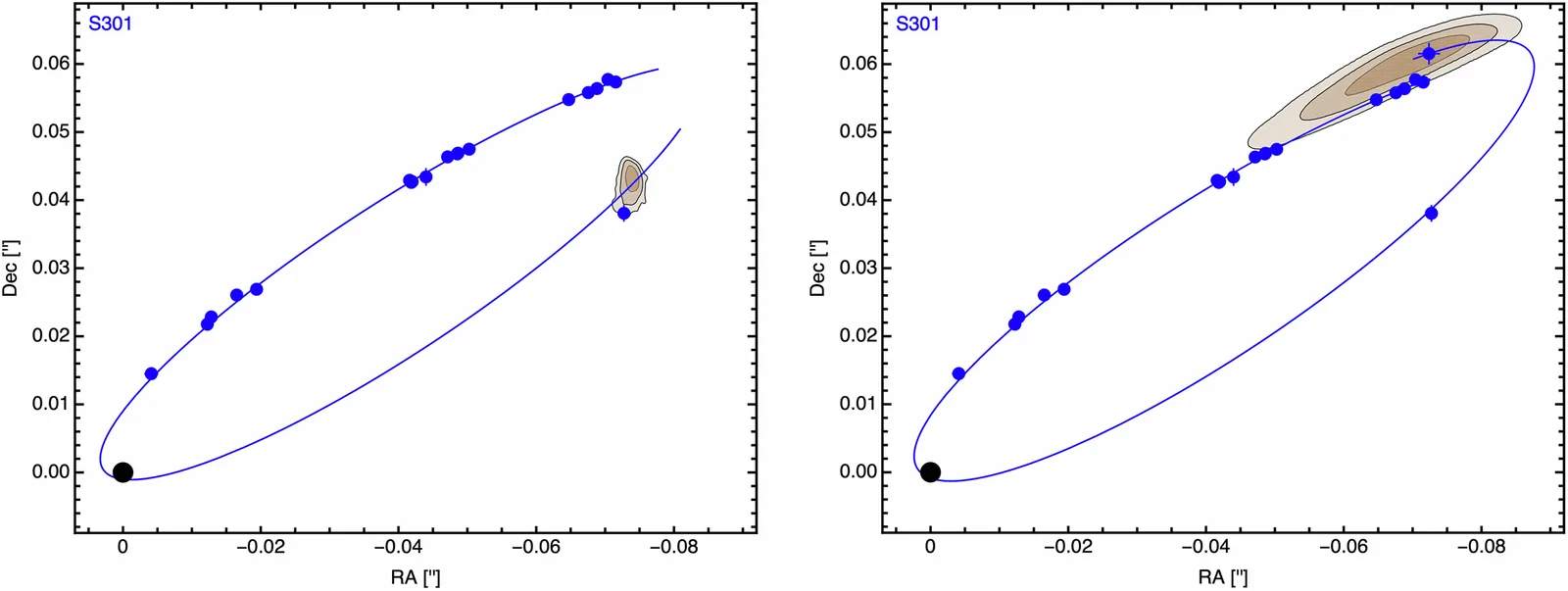
Post-dicting where S301 is expected in previous epochs. Left: Using the 2023-2025 data, we use samples from a Markov chain to predict the expected position of S301 in 2021 (brown contours). The actual inference falls close to the predicted position. Right: Using now the 2021-2025 data, we repeat the procedure to predict the star’s place in 2017. Again, we find S301 where we expect it.

**Extended Data Fig. 4:**
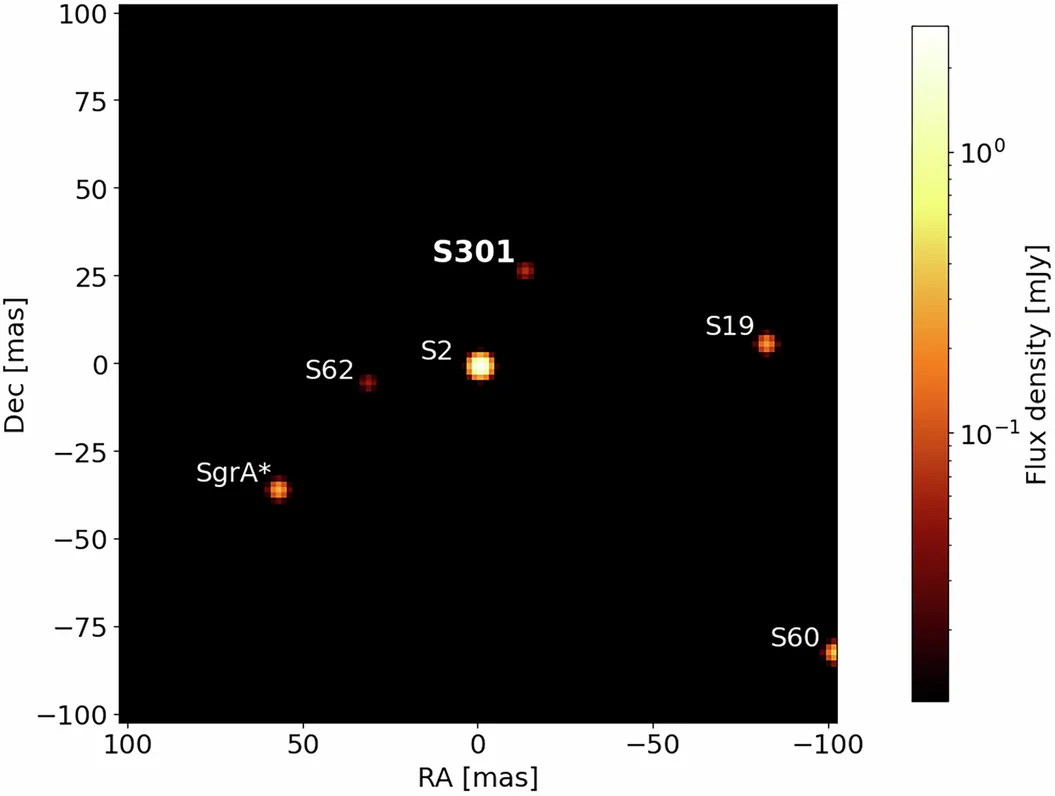
The 2017 detection of S301. G^R^ image of the data set in 2017, in which S301 is weakly inferred.

In spring 2023, we discovered a faint star 15 mas northwest of Sgr A*, which in the following months moved outwards, and which we labelled S301. From the four positions in 2023, we derived a fast (*v* ≈ (−44, +27) mas yr^−1^, |*v*| ≈ 2,000 km s^−1^) and slightly curved motion (significant at the +3/+5*σ* level), which would bring the star outside of the central field of view by 2024. We followed S301 with dedicated pointings in 2024 and 2025, yielding eight and five additional astrometric measurements, respectively. Given the preliminary orbit, we were able to post-dict the star’s positions in previous observing epochs (Extended Data Fig. 3), and whether it would be by chance in one of our pointings. We found a strong inference of S301 in 2021 and a weak inference in 2017 (Extended Data Fig. 4). These positions are less precise, as only few exposures at the respective pointings were taken. Overall, we have 19 astrometric positions of S301 that outline an ellipse on the sky and yield a consistent orbit.

We have tried to identify S301 in deep ERIS integral-field spectroscopic data, but we have not been able to detect it as a continuum source or from spectral features. Thus, we do not yet have any radial velocity information.

From the GRAVITY images, we measure the positions of S301 and Sgr A* to obtain the position vector of the star relative to the MBH. For observations, in which the S301 pointing was not centred on Sgr A* (in 2017, 2021, 2024 and 2025), the two objects are fitted relative to their respective field centres. The positional offset between the latter two is measured at the interferometric precision by the metrology system of GRAVITY. Our imaging code G^R^ also allows estimating the uncertainties in the positions, as multiple instances of the same images are inferred. We measure the brightness of the objects using the brighter sources in the images, for which photometry is available from ref. ^4^. For S301, we derive a K-band magnitude of *m*_K_ = 19.3 ± 0.3.

Using a *χ*^2^-minimization, we fit a preliminary Keplerian orbit to the astrometric data (*α*(*t*), *δ*(*t*)). According to this fit, S301 passed the pericentre of its orbit early 2023, with a three-dimensional (3D) separation significantly smaller than that of S2. Hence, we need to take into account the *β*^2^ relativistic effects, using the same model as for S2 in ref. ^8^, including the Rømer effect (retardation effects due to the finite speed of light) and Schwarzschild precession. The potential is defined by the central mass fixed with *M*_MBH_ = 4.297 × 10^6^*M*_⊙_ at a distance of *R*_0_ = 8,277 pc. We do not allow for any coordinate system offsets, as our data are interferometrically referenced directly to the near-infrared counterpart of Sgr A*.

**Extended Data Fig. 5:**
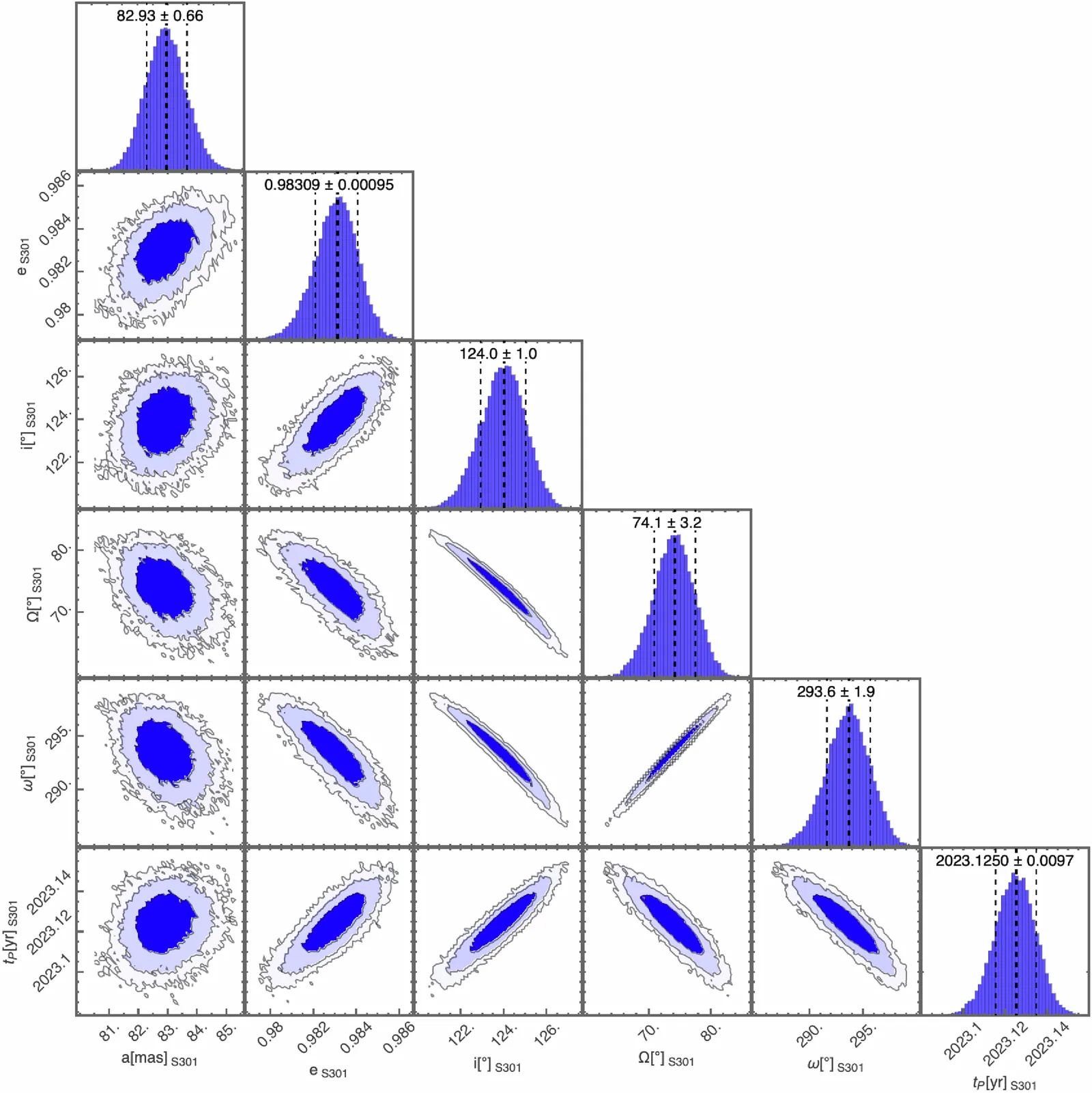
Posterior of the best-fit parameters. Posterior distribution of the six parameter orbit fit in Extended Data Table 2, left column.

Owing to the lack of radial velocity information, two equally valid solutions exist, corresponding to the two possible orientations of the orbit. In principle, the Rømer delay could break this degeneracy^23^, but the two orientations yield indistinguishable best-fit *χ*^2^ values. Except for this sign ambiguity, the orbit fit converges uniquely (as verified by sampling the posterior space with a Markov chain; Extended Data Fig. 5) at *χ*^2^ = 26 for 34 degrees of freedom. The orbit does not agree with any previously claimed detections (Methods).

**Fig. 2:**
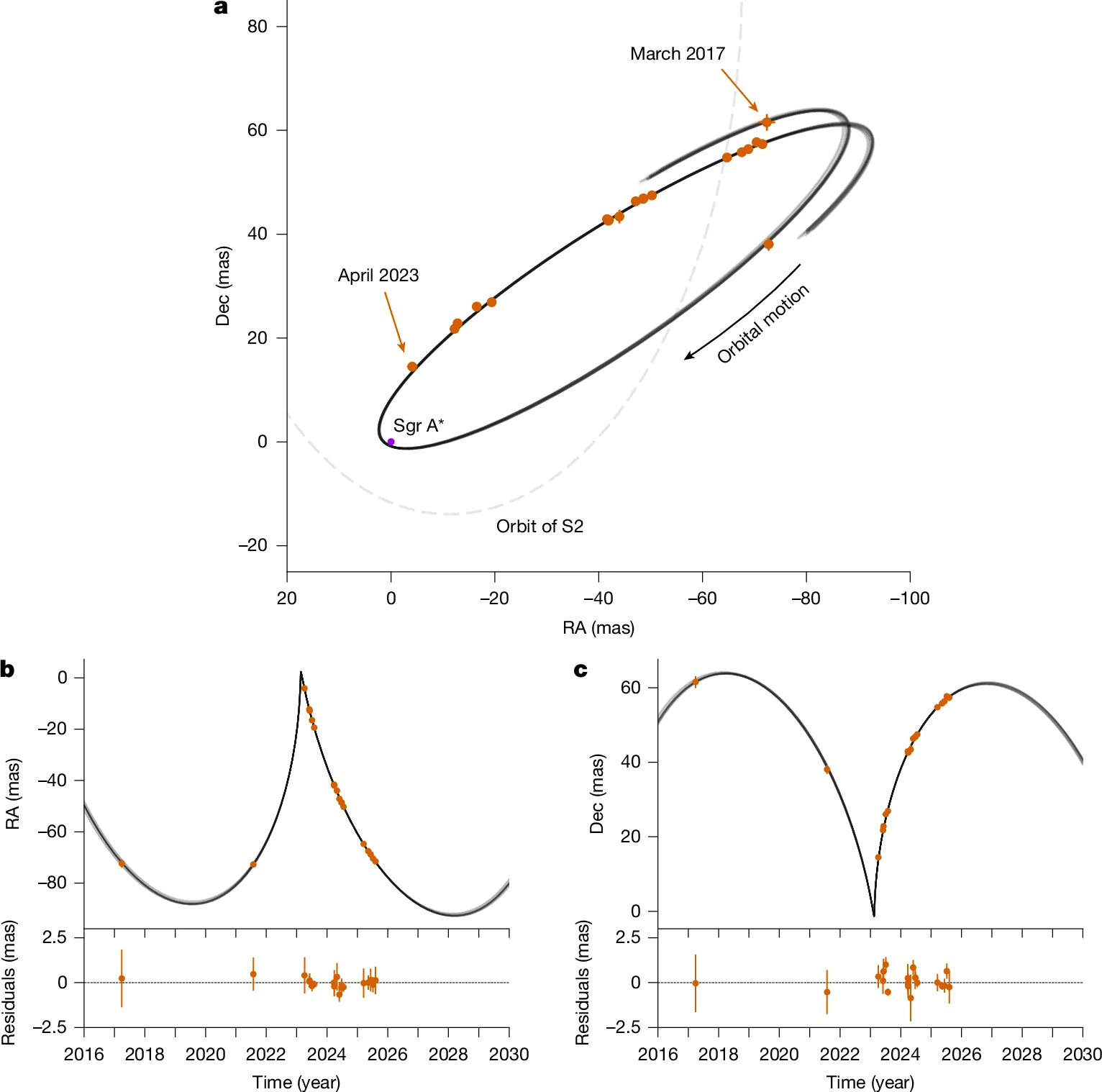
Orbit of S301 from 2016 to 2030. **a**, The best-fit relativistic orbit to the positions in a Schwarzschild metric around Sgr A* (Methods). **b**,**c**, The motion and residuals over time separately in right ascension (RA) (**b**) and declination (Dec) (**c**). Owing to the high eccentricity and short semi-major axis, the orbit visibly does not close as a result of the Schwarzschild precession.

**Extended Data Fig. 6:**
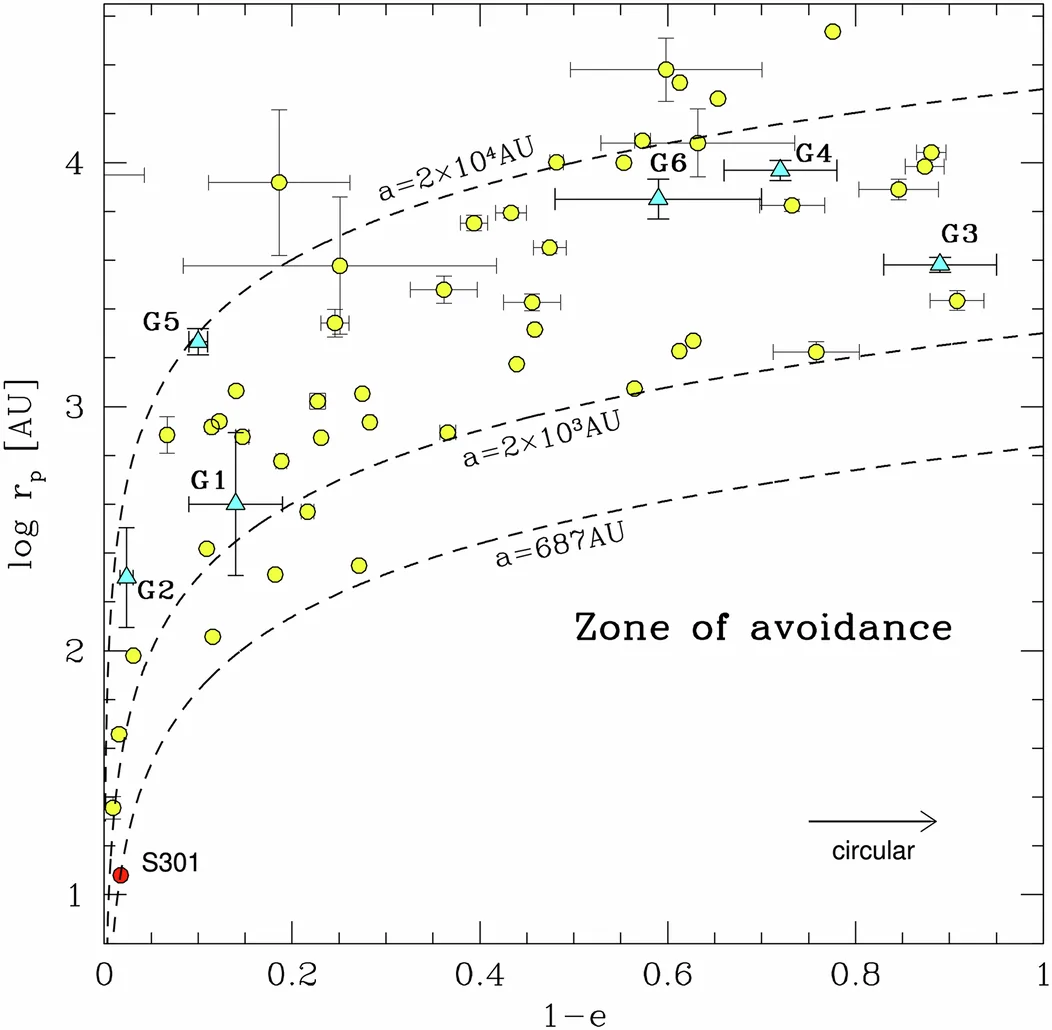
S301 and the ‘zone of avoidance’. Pericentre distance *r*_*p*_ versus (1 − *e*), with *e* the eccentricity of S-stars (yellow points) and G-clouds (cyan triangles) orbiting the central MBH^60,71^. The dashed lines connect points of constant *a*. The orbits of all stars in the lower right, empty region should have been determined by now. The lack of stars in this so-called ‘zone of avoidance’ results from the fact that no stars exist in this regime because of a correlation between semi-major axis *a* and (1 − *e*), such that stars on more circular orbits with smaller values of *e* have larger semi-major axes. S301 is depicted by the red point in the lower left corner.

**Extended Data Fig. 7:**
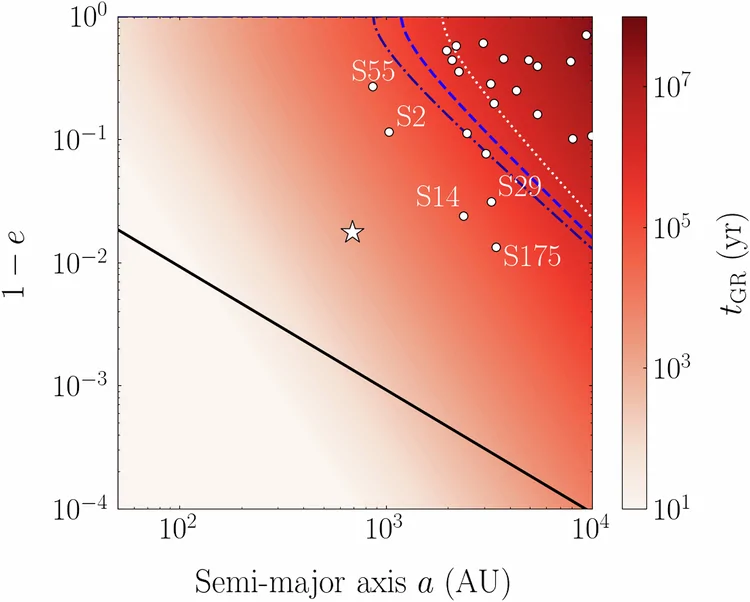
Dynamical landscape in the *a* versus (1 − *e*) phase space (in au). Left: The color map indicates *t*_GR_. The Schwarzschild Barrier (SB) boundary (*t*_GR_ = *t*_RR,*v*_) shifts significantly depending on the cusp slope *γ*. Boundaries are shown for the observed shallow cusp (*γ* = 1.3, dotted curve) and steep theoretical cusps (*γ* = 1.75, dashed curve; *γ* = 2.0, dash-dotted curve), assuming normalization at 0.25 pc. S301 (white asterisk) and other young S-stars (white circles) remain deep within the barrier in all scenarios. The solid line indicates the tidal disruption limit for S301 (1.5*M*_⊙_, 1.4*R*_⊙_).

The best-fit orbit is remarkable (Extended Data Table 2) with a semi-major axis of *a* = 83 mas (33% smaller than that of S2) and an orbital period of 8.7 years (Fig. 2). Thus, the star sets a new record for the shortest known orbital period around Sgr A* (Extended Data Fig. 6 and 7), with S55 (also called S0-102) on a 12-year orbit^24^ being the previous record holder. Even more extreme is the eccentricity of *e* = 0.9832 and 0.9821 (for the two possible orientations), leading to a pericentre distance *r*_p_ of only 136*R*_S_ and 142*R*_S_, respectively, around 10 times smaller than that of S2. This implies correspondingly stronger relativistic effects. The relativistic pericentre advance per orbit amounts to 2.0° and 1.9°, such that after just 1,560 years and 1,630 years, respectively, the orbit in its plane has revolved once. At pericentre, S301 moves with about 25,600 km s^−1^ or 8.5% of *c* and 25,000 km s^−1^ or 8.3% of *c*, respectively.

## Discussion

### Stellar type

**Extended Data Fig. 8:**
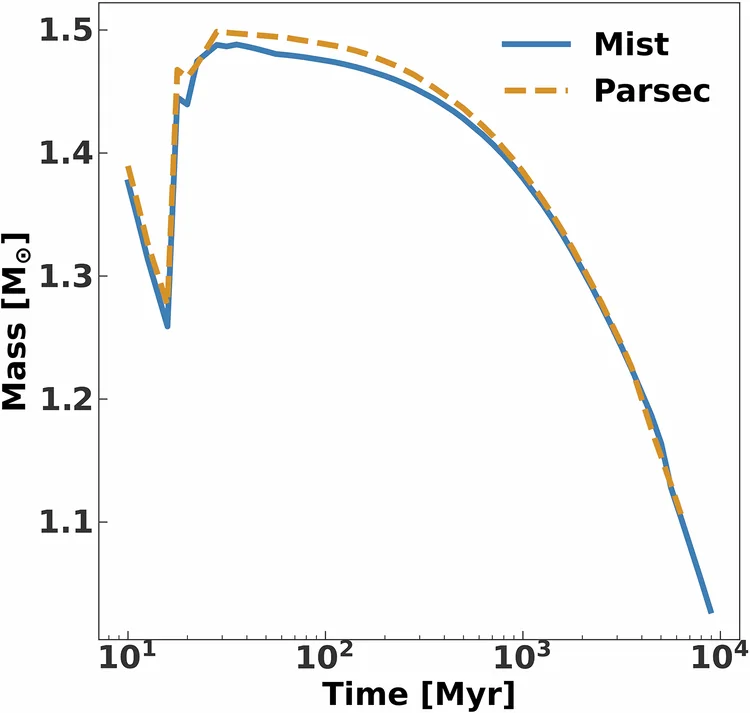
Possible ages and masses for S301. Possible ages and masses for S301, from its observed K-band magnitude and theoretical isochrones.

With *m*_K_ = 19.3 ± 0.3, S301 is too faint to be a giant in the GC, but the magnitude matches a main-sequence star of spectral type F1.5, corresponding to a mass ≲1.5*M*_⊙_ (Methods). Estimating the mass from stellar evolutionary tracks yields a consistent value (Extended Data Fig. 8). The radius of S301 is between about 1.4*R*_⊙_ and 1.6*R*_⊙_. The main-sequence life time of early F-type stars is around 2 × 10^9^ years. This interpretation is consistent with the fact that S301 did not get tidally disrupted at pericentre. A giant star with r_*_ = 0.5 au and m_*_ = 3*M*_⊙_ begins to lose a fraction of its envelope at its tidal radius ${r}_{{\rm{t}}}={r}_{* }{({m}_{* }/{M}_{\mathrm{MBH}})}^{1/3}\approx 500\,{R}_{{\rm{S}}} > {r}_{{\rm{p}}}$. The loss of mass would affect the orbit at pericentre, which is not observed. By contrast, for a main-sequence star *r*_t_ ≈ 10 *R*_S_ < *r*_p_, such that it is safe from tidal disruption (and tidal heating; Methods).

Spectrally, we therefore expect S301 to show exclusively the Brackett-*γ* absorption line, but no further features in the infrared K-band^25^. Our current ERIS spectroscopy is not deep enough to test this. Future MICADO^26^ spectroscopy at the ELT will have no problem in seeing the spectral features and will be able to determine the radial velocity of S301.

### Spin sensitivity

**Fig. 3:**
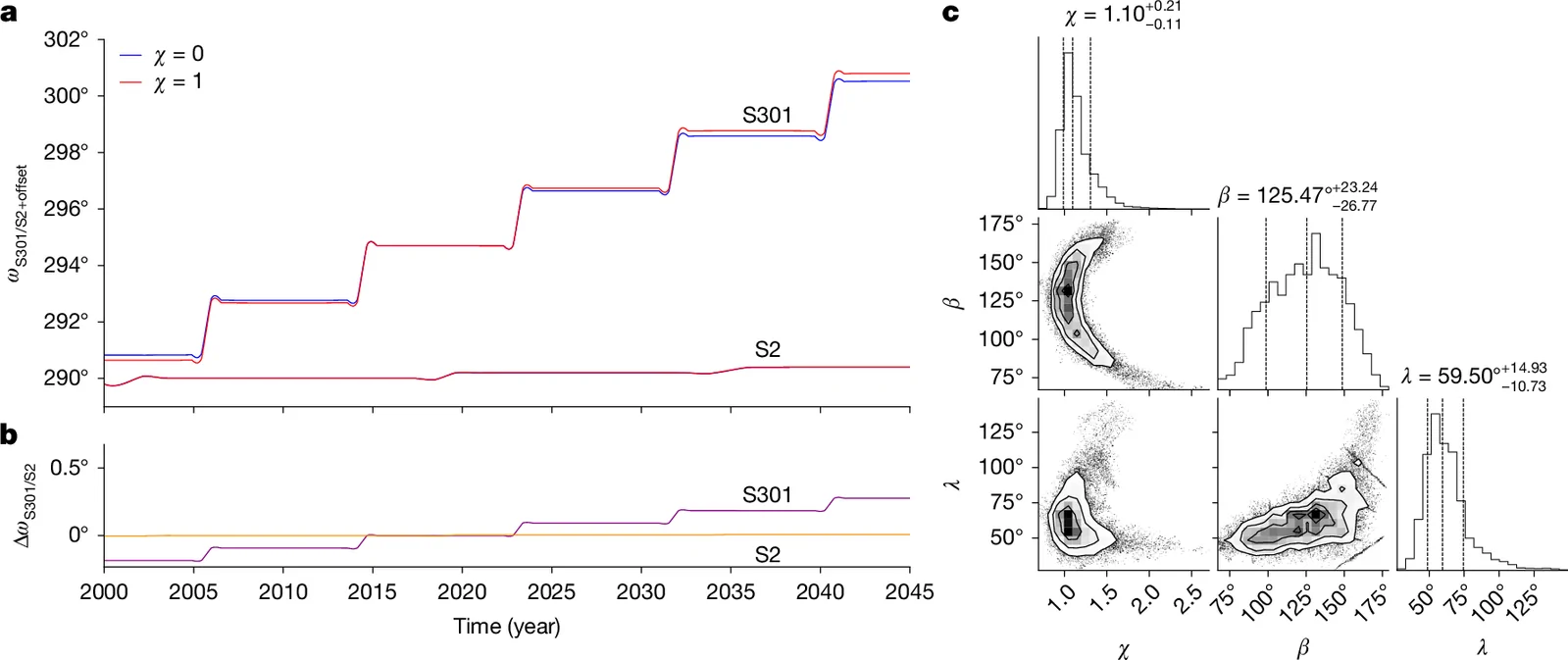
Sensitivity of the S301 orbit to the spin of Sgr A*. **a**, Change of the orientation of the orbital ellipse (measured by the osculating longitude of pericentre *ω*) as a function of time for S301 in comparison with S2. The discrete steps occur when the respective star passes the pericentre of its orbit. The blue lines are for a non-rotating MBH (*χ* = 0), the red ones correspond to a randomly oriented (*λ* = *ξ* = 0, pointing towards the Sun), maximally spinning (*χ* = 1) black hole. The extra-precession due to the spin of Sgr A* of the S301 orbit is comparable to the changes induced by the 1PN-terms of the Schwarzschild metric of S2, which GRAVITY has detected already^5,8^. **b**, The difference in *ω* between the two spin values. **c**, Example of constraints on the spin parameters (*χ*, *λ*, *ξ*) for a mock dataset with future simulated data up to 2035. These mock data constrain *χ* with an uncertainty <0.2 and also the orientation to around ±30°.

**Extended Data Fig. 9:**
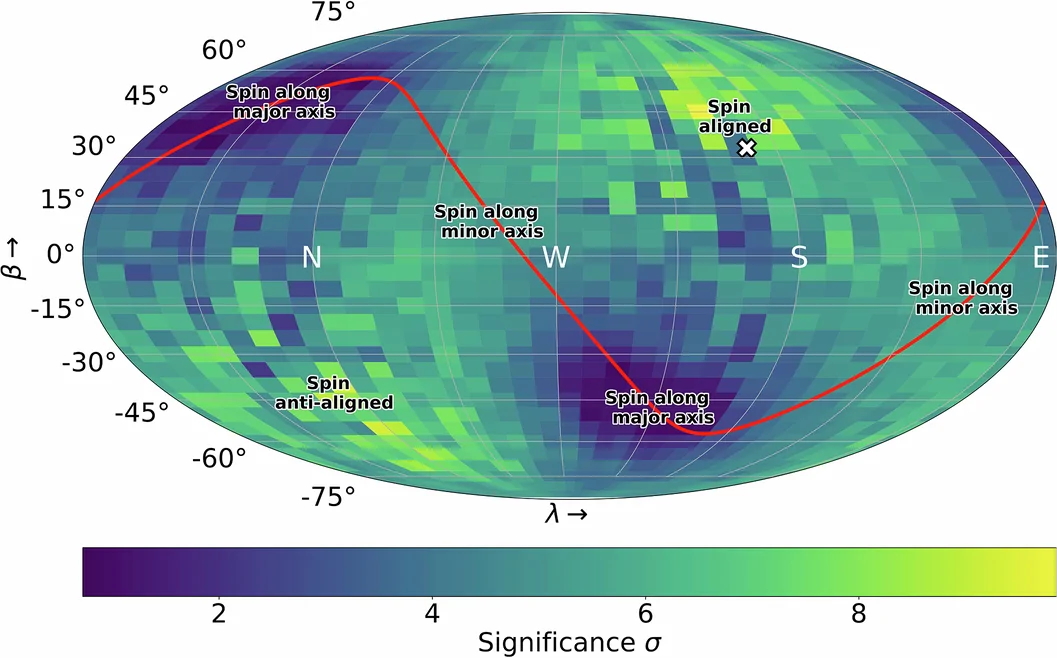
Sensitivity of the S301 orbit to the spin orientation. Significance of the detection of the spin as a function of orientation of the spin vector for our simulated data with *χ* = 1. Over a wide range of possible orientations, we expect to be able to detect the spin. The cross marks the orientation of the orbital angular momentum vector of S301. The red line corresponds to maximum misalignment between black hole spin and orbit. The best chances of detection are for co- or anti-alignment.

The most exciting aspect of the orbit of S301 is its extremely small pericentre distance, such that it is sensitive to the spin of Sgr A* to a degree accessible within around a decade, using existing and/or planned instrumentation. Figure 3a,b predicts for a maximally rotating black hole (*χ* = 1; with $\chi \equiv cJ/(G{M}_{\mathrm{MBH}}^{2})$ the dimensionless spin parameter) the in-plane precession effects. The dominant effect is the Schwarzschild precession, advancing the pericentre by 1.9º per revolution. The in-plane contribution of the Lense–Thirring precession is $0.1{1}^{^\circ }\chi \cos \xi$ per revolution (where *ξ* is the inclination between spin axis and orbital angular momentum; Methods), comparable to the angle by which S2′s pericentre advances due to the Schwarzschild metric, and which we have measured in ref. ^8^. The time scale for the spin precession^27^ is about 58,000 years. Forecasting future observations allows testing by when the data would be measurably sensitive to the spin. Using reasonable assumptions, we estimate that within a decade, there is a fair chance to directly measure the spin of Sgr A* from the S301 orbit (Fig. 3c, Methods and Extended Data Fig. 9).

S301 provides one of the first and currently only practical ways to measure the spin of Sgr A* with stellar dynamics. Although all other known S-stars owed to their larger pericentre distances are insensitive to frame dragging on observationally accessible time scales, they will provide constraints on *M*_MBH_ and *R*_0_, which can be used as priors, reducing degeneracies and assisting the spin measurement. These stars would also constrain Newtonian perturbations due to dark masses orbiting Sgr A*, which are expected to be sub-dominant. In the longer term, S301 opens a path towards constraining the quadrupole moment of Sgr A* and testing the Kerr no-hair relation^28^ once sufficiently long time series of sufficiently accurate astrometry and spectroscopy become available.

The Schwarzschild precession of S301 offers the opportunity to tighten constraints on possible deviations from general relativity (for example, strong-field parametrized post-Newtonian (PPN) parameters) and on a putative extended mass distribution. The current dataset is consistent with general relativity at the first post-Newtonian (1PN) level, although the resulting PPN bounds are weaker than those derived from S2 (refs. ^8,29^) with *f*_SP_ = 0.94 ± 0.88. A joint analysis may improve these constraints, and continued monitoring of S301 will significantly strengthen them.

### Origin

Another interesting aspect of the orbit is its history, which we can constrain from the dynamical timescales (Methods and Extended Data Table 3). We see that the star and its orbit can be affected by stellar collisions and relaxation. As star formation so close to the MBH is unlikely given the strong tidal effects, S301 has likely formed elsewhere and migrated to its current location. Given its mass, the life time of S301 is comparable to the two-body relaxation time, and it is therefore unlikely to have formed in the nuclear cluster and slowly migrated inwards through two-body relaxation. Instead, the extreme value *e* ≃ 0.98 is naturally produced in a Hills disruption of a compact main-sequence binary by the MBH^30^ (Methods).

In the Hills picture, the timescale hierarchy at the radius of S301 constrains its origin and evolution further. The angular momentum relaxation and collision time scales are shorter than the main-sequence lifetime. If the star had been in the GC for more than about a few ×10^7^ years, its eccentricity would have thermalized, losing memory of its initial condition. In this case, the high eccentricity simply occurs by chance (there is an approximately 3% chance to find such a high eccentricity for a thermal distribution). Alternatively, S301 may have been injected into the GC recently, so that it has not had time to relax, and constitutes a pristine fossil of its capture event.

The long two-body relaxation and gravitational wave inspiral times imply that the semi-major axis of the star has not evolved significantly over its lifetime, and thus its present-day orbit constrains the progenitor binary. The measured semi-major axis is related to one of the pre-disruption binary, yielding *a*_bin_ ≈ 0.1 au (Methods). These compact binaries are common among F-type stars^31^ and are expected to be tidally circularized and nearly synchronized. S301 would then have an equatorial rotation velocity *v*_rot_ ≈ 20–70 km s^−1^, which might be observable with future high-resolution spectroscopy from ELT/MICADO, providing a direct test of the Hills origin. Taken together, the properties of S301 suggest a simple and self-consistent picture: a compact main-sequence binary was tidally separated by Sgr A*, leaving behind S301 on the most relativistic stellar orbit known and ejecting its companion as a hyper-velocity star.

## Methods

### Details of observations

Our observations, from which we inferred S301, comprise 19 datasets spanning more than 8 years. We first noted S301 in 2023, when pointing directly to Sgr A*. Given the position of the star close to Sgr A* and its large proper motion, it was clear that it might be on a tight orbit, and we followed up with dedicated observing campaigns over the course of the next years. We summarize the data used for this work in Extended Data Table 1.

Based on the orbital coverage from 2023 to 2025, we were able to trace S301 back in time and identified two previously acquired datasets in 2017 and 2021, in which the star should be present. For this, we fit a preliminary orbit to the 2023–2025 data and used samples from a Markov chain to predict where the star should be in 2021. The dataset in 2021 was acquired during an observation that involved multiple other pointings in the GC to create a mosaic of the central about 200 × 200 mas (ref. ^22^). This particular pointing was affected by bad seeing conditions, however, and we apply stricter cuts and accept as a post-processing step only fringe-tracking ratios of 90% for the individual detector integrations, effectively discarding data with low coherent flux. This procedure led to the inference of S301 in this particular epoch, close to the predicted position (Extended Data Fig. 3, left).

With that position in hand, we repeated the orbit fit and used the updated Markov chain to predict the S301 position in 2017. The 2017 data were acquired as part of the monitoring of the motion of the star S2, an object around five magnitudes brighter than S301. The dataset consists of individual exposures across five consecutive nights in March 2017, partially recorded in split-polarization mode. In the image reconstruction of this dataset, we treat both polarizations in polarized exposures as individual measurements and combine them with the unpolarized data. Again, S301 is found close to the predicted place (Extended Data Fig. 3, right).

### Highest-resolution, deep images of the central 800 au

The high-angular-resolution, high-fidelity images of the GC are reconstructed from GRAVITY data, an example of which is shown in Extended Data Fig. 1 with the image reconstruction tool GRAVITY-RESOLVE (G^R^)^22^. This code is designed to reconstruct stars in the GC that appear to GRAVITY as unresolved point sources and, in particular, to find faint, yet undiscovered stars. G^R^ is based on a hierarchical forward model that incorporates the instrument response of GRAVITY, including both optical aberrations and the spectral transmission within the beam combiner, as well as a statistical model of the GC. The intrinsic multitude of degrees of freedom of an image is tamed with Bayesian inference, in which the statistical model of the GC provides the previous information. With G^R^, we exploit supreme imaging resolution of ≃ 1.7 mas of GRAVITY and its phase-referencing abilities, which allow for high contrast images and routine mosaicking as pioneered in radio interferometry (F. Mang et al., manuscript in preparation).

The individual positions of S301 over time, as shown in Fig. 2, were inferred in a first step from imaging. We initialized the model in G^R^ by invoking all known, bright sources within the field of view. Positions and fluxes of the stars and Sgr A* are constrained by previous distributions that reflect current knowledge. We applied G^R^ a total of 10 times to the same dataset with varying initial random seeds. Tentative faint sources that may appear in the image of individual reconstructions are accepted only when they are inferred in at least 5 out of 10 reconstructions. Their corresponding positions in the image grid are then referenced to Sgr A* and subsequently averaged.

### Astrometric errors

The uncertainties of the newly inferred sources are derived from the scatter over the different runs, also taking into account the finite resolution of the pixel grid. This is an improvement over^22^ where the errors were simply approximated by the size of the pixels in the image. If the same faint source is inferred in the same pixel for the 10 individual runs, the corresponding standard deviation is zero, albeit unphysical. Hence, the discretized position space needs to be considered when stating errors on astrometry. Instead of quantifying the error based on the number of same-pixel inferences in a set of detections, we opt for a general approach, which may be conservative, but prevents an underestimation of errors. We derive a discretization error by calculating the root mean square error for a single pixel within the image for both directions, right ascension and declination, independently. Considering a pixel size of 0.8 mas per pixel, this evaluates to about 207 μas and represents the statistical uncertainty in astrometry originating from the pixel grid in G^R^ images. This value acts effectively as a noise floor.

### Fitting of GRAVITY data

Apart from imaging with G^R^, we apply two other tools to analyse GRAVITY data. These methods serve to fit individual stars, that is, parameterized point sources, foremost to determine their astrometry and photometry. They allow for a crucial cross-check whether S301 is inferred at the same position with a comparable photometry as with the image reconstruction. Both fitting tools are separately implemented in different programming languages.

In a first step, every GRAVITY exposure is fit separately to infer the variable flux of Sgr A*, equivalent to determining a light curve over time. Both inferred fluxes and positions of Sgr A* and other bright, known sources then provide the starting values for a subsequent combined fit. For S301, the values from the imaging code are used as starting values. S301 is inferred by both methods at the same position as with the imaging code, within statistical errors.

Although the fitting codes yield the same positions for S301 as G^R^, it would be de facto impossible to find a star such as S301 using just a fitting code. The fitting essentially returns a local minimum, whereas the imaging efficiently explores the full parameter space.

### Imaging with CLEAN

We also inferred S301 with a more classical imaging routine, namely, CLEAN (Extended Data Fig. 2). For that, we first CLEANed on Sgr A* in the individual exposures to capture its variability. The corresponding coherent flux is then subtracted from the data before combining the data and running a deeper CLEAN, allowing to infer fainter sources, such as S301 and S62.

### Best-fit orbit

We fit the astrometric positions of S301 in the same way as we did for the S2 data in ref. ^8^, taking into account Rømer delay and the 1PN correction of the motion due to the Schwarzschild nature of the gravitational potential. Relativistic Doppler effect and gravitational redshift do not matter as they only affect radial velocity measurements, which we currently do not have. This lack also results in an ambiguity in the 3D orientation of the orbit. The two viable orbit solutions to the proper motion of S301 are given in Extended Data Table 2 (angle conventions follow ref. ^4^) and leave the semi-major axis, eccentricity and time of periastron unchanged within errors. In principle, the Rømer delay could break the ambiguity^23^, but does not yet for the limited phase coverage of S301. The orbit stands out compared with other S-stars. For an illustration, see Extended Data Figs. 6 and 7. The high eccentricity of S301, combined with the small value of the semi-major axis, makes S301 an outlier with the smallest value of *r*_p_ = *a*(1 − *e*). Also note that the orbital plane of one of the two possible solutions agrees to within 3° with the inner clockwise disk of young, massive stars in ref. ^32^.

### Measuring the spin of Sgr A* with S301

The orbit-averaged Lense–Thirring effects for the in-plane and out-of-plane major axis precession are, respectively,^33,34^

1 $$\begin{array}{c}\Delta {\varpi }_{\mathrm{LT}}=-8{\rm{\pi }}\chi \cos \xi {\left(\frac{G{M}_{\mathrm{MBH}}}{a(1-{e}^{2}){c}^{2}}\right)}^{3/2}\,\,,\\ \Delta {\Theta }_{\mathrm{LT}}=-4{\rm{\pi }}\chi \sin \xi \sin \lambda {\left(\frac{G{M}_{\mathrm{MBH}}}{a(1-{e}^{2}){c}^{2}}\right)}^{3/2},\end{array}$$

where *ξ* is the inclination between spin axis and orbital angular momentum, and *λ* is the position angle of the projection of the spin axis onto the orbital plane. For S301, the in-plane contribution per revolution amounts to 0.11°*χ* cos *ξ* .

To assess how S301 probes the spin of Sgr A*, we perform a mock data analysis combining current and simulated future observations. We construct synthetic astrometric and radial-velocity datasets extending the existing measurements with simulated observations between 2026 and 2035, using the values of the orbital parameters given in Extended Data Table 2, with mass and distance of Sgr A* from ref. ^5^. We optimistically adopt *χ* = 1 and an orientation approximately aligned with the stellar orbital angular momentum. In this case, the Lense–Thirring effect produces mainly in-plane precession and little precession of the orbital plane. The resulting mock data are fitted to test whether the spin parameters can be recovered. We assume a realistic astrometric precision of 100 μas (as expected for the final performance of GRAVITY+) and 1 km s^−1^ accuracy on the radial velocity as reachable with future ELT/MICADO observations. A sampling of 10 data points per year until 2035, with 10 additional data points around pericentre, would yield a spin constraint with an uncertainty on *χ* of <0.2 (Fig. 3c), equivalent to a >5*σ* detection of *χ* = 1 compared with the non-spinning (*χ* = 0) case. In Extended Data Fig. 9, we show the dependence of the significance of the spin detection in our simulations as a function of orientation of the black hole spin. The maxima of significance correspond to alignment and anti-alignment of the spin and orbital angular momentum, whereas the minima correspond to alignment of the spin along the semi-major axis of the orbit. A more detailed discussion is provided in another study (K.A.E.D. et al., manuscript in preparation). Also, we note that future robust spin constraints will require modelling up to 2PN (second post-Newtonian) order to avoid systematic biases at low spins.

### Spectral type, mass and age of S301

Given the magnitude *m*_K_ = 19.3 ± 0.3 and assuming an extinction of 2.42 (ref. ^35^) and a GC distance of *R*_0_ = 8.3 kpc (ref. ^23^), S301 has an absolute K magnitude of about 2.28. It is too faint to be a giant, but it instead is compatible with being a main-sequence star. Its spectral type then is a late A-type or an early F-type star (see also figure 2 in ref. ^36^), which means its colour index is *V* − *K* = 0.6 (ref. ^37^). With an absolute V magnitude of 2.88, it has *L* = 5.5 *L*_⊙_ and a spectral type of F1.5, corresponding to a mass of just below 1.5*M*_⊙_. Alternatively, its mass can be estimated to be between approximately 1.1*M*_⊙_ and 1.5*M*_⊙_, depending on the age of the star, with younger ages corresponding to larger masses.

We infer the possible ages and masses of this star, using the observed brightness and the MIST^38–40^ isochrones (MIST v.1.2 tracks with *Ω*/*Ω*_crit_ = 0.4 and solar metallicity). Specifically, we identify evolutionary points at which the track magnitude crosses the inferred absolute magnitude. The stellar ages and masses for these points are shown as a solid, blue line in Extended Data Fig. 8. Note that the MIST tracks do not include the K band magnitude, and we use the JWST F210M magnitude as a proxy.

To test the robustness of our results, we repeat this analysis with the K band magnitude from PARSEC isochrones (v.1.2S; refs. ^41–45^) and show the possible ages and masses in Extended Data Fig. 8 (dashed, orange line). We note that unlike MIST, the PARSEC tracks do not include stellar rotation. Nonetheless, the results are in excellent agreement with the stellar mass ranging from about 1.1*M*_⊙_ to 1.5*M*_⊙_, depending on the age of the star.

### Tidal effects

A previous study^46^ suggests that tidal effects prevent observations of Kerr effects around Sgr A*. However, they consider stars 10*M*_⊙_ or heavier. S301 is sufficiently small so that these tidal effects are negligible. The energy input per orbit can be estimated, using the ref. ^47^ formalism

2 $$\begin{array}{c}\Delta E\approx {T}_{2}(\eta ){\left(\frac{{M}_{\mathrm{MBH}}}{{m}_{\star }}\right)}^{2}\frac{G{m}_{\star }^{2}}{{R}_{\star }}{\left(\frac{{r}_{{\rm{p}}}}{{R}_{\star }}\right)}^{-6},\\ \eta ={\left(\frac{{m}_{\star }}{{M}_{\mathrm{MBH}}}\right)}^{1/2}{\left(\frac{{r}_{{\rm{p}}}}{{R}_{\star }}\right)}^{3/2},\end{array}$$

where *r*_p_ is the pericentre distance, *R*_⋆_ is the stellar radius, *m*_⋆_ is the stellar mass and *T*_2_ is the tidal coupling constant. Here, we include only the leading-order quadrupole term. We estimate the tidal coupling constant using the fits from ref. ^48^, assuming an *n* = 3 polytrope. Note that these fits extend only to *η* = 10, and we extrapolate them using the logarithmic slope there. Thus,

3 $$T(\eta )\approx 3.8\times 1{0}^{-5}{\left(\frac{\eta }{10}\right)}^{-6.5},$$

for *η* ≥ 10. This yields $\delta E\approx 1{0}^{-16}G{m}_{\star }^{2}/{R}_{\star }$. It would take an order of 10^17^ years for tides to inject an order of unity fraction of the energy of the star. Therefore, tidal heating can be neglected at the current orbit of the star.

### Comparison with previously claimed short-period stars

Over the past 5 years, several short-period stars with orbital periods as low as 4 years were claimed to have been discovered by one team using adaptive-optics based imaging techniques^49,50^, thus at a resolution 15 times worse than the GRAVITY data. Safely, we can exclude that the objects in ref. ^49^ named S4711 and S62 (different from the star our team calls S62; see ref. ^51^), and that have similar orbital periods as S301, are actually S301:

- The eccentricity of S4711 with *e* = 0.768 is considerably less than that of S301, and the claimed orbit has a different projection on sky, extending towards the East.
- Although S62 features a comparable eccentricity of *e* = 0.976, the claimed orbit revolves anticlockwise, unlike S301.

Apart from this, none of the other claimed stars have orbital elements similar to the ones of S301. Hence, S301 is newly discovered.

Furthermore, it is worth noting that the limiting magnitude of *m*_K_ ~ 20 inferred from the GRAVITY observations and reported here should have allowed us to easily detect any of the claimed discoveries if covered in our exposures. In none of our reconstructed images, we have detected a significant contribution of flux beyond background noise that we could attribute to the claimed stars.

### Newtonian perturbations of the spin measurement

The simulations for the spin measurement assume that S301 orbits an isolated Kerr black hole in the absence of external perturbations. As noted in ref. ^33^, gravitational perturbations from a population of stellar-mass black holes, expected to form a power-law-density stellar cusp around Sgr A*, can induce orbital precession comparable in magnitude to the Lense–Thirring effect, thereby complicating the measurement of the spin. Recent constraints from ref. ^29^ limit the extended mass within the central 10 mpc to ≲1,200*M*_⊙_. To assess the impact of a realistic perturber population, following ref. ^52^, we performed *N*-body simulations of S301, including a cluster of 60 stellar-mass black holes of 20*M*_⊙_ each, distributed within 10 mpc of Sgr A* according to a density profile *ρ*(*r*) ∝ *r*^−2^. Considering 100 independent realizations of the initial conditions, we find that the cluster induces an average orbital-plane precession per orbital period of $0.6{5}_{-0.56}^{+0.36}\,\mathrm{arcmin}$. This contribution is typically sub-dominant compared with the Lense–Thirring precession for moderate-to-high dimensionless spins and favourable orientations of the black-hole spin relative to the orbital angular momentum. Importantly, the Lense–Thirring effect is concentrated near pericentre, whereas perturbations from a granular stellar background tend to produce their largest observable deviations near apocentre^52,53^. This phase separation, together with the distinct temporal signatures of the two effects, offers a promising avenue to disentangle the relativistic spin signal from stellar perturbations and thereby enable a robust measurement of the MBH spin.

In an extreme case in which all the mass (allowed by S2 observations) is concentrated within the orbit of S301 in a disk and in which the orientation of the orbit is a few degrees from the disk, the nodal precession due to the disk might be larger, by up to an order of magnitude, than the Lense–Thirring precession. However, for most orientations and for less extreme mass distributions, the Newtonian effect of this mass on the nodal precession is expected to be one or two orders of magnitude smaller than the Lense–Thirring effect.

### A Hills origin for S301

For the Hills mechanism^30,54–58^, one of the binary components is captured at a semi-major axis of

4 $${a}_{{\rm{cap}}}={f}_{1}{\left(\frac{{M}_{{\rm{MBH}}}}{{m}_{{\rm{bin}}}}\right)}^{2/3}{a}_{{\rm{bin}}},$$

where *f*_1_ ≈ 0.5 for circular, equal mass binaries^59^. The captured star inherits the pericentre of the orbit of the binary around the MBH, and so the binary separation must be no more than a factor of few times ${({M}_{{\rm{MBH}}}/{m}_{{\rm{bin}}})}^{1/3}{a}_{{\rm{bin}}}$ (the characteristic binary tidal disruption radius). Thus, the eccentricity of the captured star will be

5 $${e}_{{\rm{cap}}}=1-{f}_{2}{\left(\frac{{m}_{{\rm{bin}}}}{{M}_{{\rm{MBH}}}}\right)}^{1/3},$$

where *f*_2_ is at most a factor of order unity. The expected distribution of *f*_2_ and hence of *e*_cap_ will depend on the distribution of binary properties (for example, the mass ratio, inclination, internal eccentricity) and the pericentre distribution of disrupting binaries. A recent study^60^, simulated binary disruptions in the GC for an observationally motivated binary population, assuming a full loss cone and an isotropic inclination distribution. For captured stars with semi-major axes between 2 × 10^−3^ pc and 4 × 10^−3^ pc and masses between 1.3*M*_⊙_ and 1.7*M*_⊙_ the eccentricities ranged between 0.97 and 0.996 (5th–95th percentile) with a median eccentricity of 0.985. Thus, binary disruption would naturally explain the observed eccentricity of the star.

The measured semi-major axis constrains the pre-disruption binary. For nearly equal masses, the Hills mapping gives ${a}_{{\rm{cap}}}\approx \frac{1}{2}$ ${({M}_{{\rm{MBH}}}/2{m}_{* })}^{2/3}{a}_{{\rm{bin}}}$ (ref. ^59^). Identifying *a*_cap_ ≃ *a* and adopting m_*_ ≃ 1.3–1.7*M*_⊙_ yields a pre-disruption separation *a*_bin_ ≈ 0.05–0.2 au, corresponding to orbital periods *P*_bin_ ≈ 5–20 days. We find a similar range of progenitor semi-major axes in the simulations of ref. ^60^, with an observationally motivated mass ratio distribution.

These compact binaries are common among F-type stars^31^ and are expected to be tidally circularized and nearly synchronized. If S301 was initially synchronized, its spin period at capture would have been comparable to *P*_bin_, implying an equatorial rotation velocity *v*_rot_ ≈ 20–70 km s^−1^ for R_*_ ≃ 1.3–1.7*R*_⊙_. High-resolution spectroscopy with ELT/MICADO might therefore provide a direct, testable prediction of the Hills-binary origin using a measurement of vsini.

Hills events simultaneously populate the innermost S-star cluster and the halo hyper-velocity star population. For Hills disruption rates of order ${\dot{N}}_{{\rm{H}}{\rm{i}}{\rm{l}}{\rm{l}}{\rm{s}}}\approx 1{0}^{-5}-1{0}^{-4}\,{{\rm{y}}{\rm{r}}}^{-1}$, simple steady-state arguments suggest that we expect of a order of a few S301-like stars on similarly relativistic orbits at any given time. The Hills disruption rate is supported by both the observed number of hyper-velocity stars in our galaxy^61^ and theoretical estimates as well as the observed tidal disruption rate in other galaxies. With roughly 10% of them having the right separation range to produce S301-like orbits (see equation (4) above), we take the formation rate of stars on S301-like orbits to be ${\dot{N}}_{S301}\approx 1{0}^{-6}\,{{\rm{y}}{\rm{r}}}^{-1}$. Given the collisional lifetime of more than 10^8^ years (Extended Data Table 3), we expect in steady state about a hundred stars with such an orbit. Most of them are likely solar mass stars, and hence still too faint to be detected. Further analysis of the observed coverage and current sensitivities of GRAVITY+ is needed to assess if this estimate is consistent with only one S301-like object detected so far; and continued GRAVITY+ monitoring and future ELT observations may therefore reveal a population of faint, low-mass S-stars deep in the potential well, enabling ensemble constraints on the spin of Sgr A* and on the distribution of stellar and remnant perturbers in the innermost approximately 10^−2^ pc.

### Dynamical time scales for S301

In this section, we will estimate the time scales for the orbital relaxation and collisions for S301.

#### Relaxation times

Relaxation is the dynamical evolution of objects due to perturbations from their environment. In the GC, relaxation can be subdivided into resonant and non-resonant parts. Resonant relaxation corresponds to the evolution of stellar orbits due to coherent torques from the background. This can lead to rapid evolution of angular momentum. On longer time scales, non-resonant (two-body) relaxation evolves the orbital energy due to uncorrelated two-body encounters (see ref. ^62^ for a review).

The two-body relaxation time scale is approximately (see equation 5.61 in ref. ^63^)

6 $${t}_{\mathrm{rx}}=0.34\frac{{\sigma }^{3}}{{G}^{2}n\langle {m}^{2}\rangle \mathrm{ln}\varLambda },$$

where $\sigma \approx \sqrt{GM/(1+\gamma )/r}$ is the one-dimensional velocity dispersion, *n* is the number density (with power-law index −*γ*), and ⟨*m*^2^⟩ is the second moment of the mass function. Owing to the quadratic mass dependence, the most massive species (namely, stellar mass black holes) often dictate this time scale.

We follow ref. ^64^ to estimate the background density profile. We model the background as two species: 10*M*_⊙_ black holes and 1*M*_⊙_ stars. This is a reasonable approximation for modelling relaxation in an evolved galactic nucleus and has been used extensively in the literature^63^. The stars are initialized with a Nuker density profile^65^, namely,

7 $$\rho (r)={\rho }_{{\rm{o}}}{\left(\frac{r}{{r}_{{\rm{o}}}}\right)}^{-\gamma }{\left[1+{\left(\frac{r}{{r}_{{\rm{o}}}}\right)}^{\alpha }\right]}^{(\gamma -\beta )/\alpha },$$

with *γ* = 1.5, *α *= 2 and *β* = 5. The density normalization is set by the total mass: 2.5 × 10^7^*M*_⊙_ and 2.5 × 10^5^*M*_⊙_ for the stars and black holes, respectively. The central MBH starts at 4.15 × 10^6^*M*_⊙_ and grows to 4.27 × 10^6^*M*_⊙_ by consuming stars and black holes. The profile is allowed to relax for 10 Gyr, such that the final density profiles are not too sensitive to the initial conditions. In the end, the total mass within 0.01 pc is approximately 1,200*M*_⊙_, consistent with the latest constraints on the enclosed mass within the apocentre of S2 (ref. ^29^). Furthermore, the final stellar density at 1 pc (approximately 9 × 10^4^*M*_⊙_ pc^−3^) is comparable to observational estimates^66^.

The following power-law fits approximate the final density profile between about 10^−4^ pc and 0.01 pc:

8 $$\begin{array}{c}{\rho }_{\mathrm{bh}}=4.95\times 1{0}^{8}{\left(\frac{r}{0.0033\mathrm{pc}}\right)}^{-1.75}{M}_{\odot }\,{\mathrm{pc}}^{-3},\\ {\rho }_{\ast }=3.46\times 1{0}^{8}{\left(\frac{r}{0.0033\mathrm{pc}}\right)}^{-1.4}{M}_{\odot }\,{\mathrm{pc}}^{-3}.\end{array}$$

Combining equations (6) and (8), we find that the two-body relaxation time scale at the semi-major axis of S301 (3.3 × 10^−3^ pc) would be about 9 × 10^8^ years.

To estimate the angular momentum relaxation time, including resonant relaxation, we follow ref. ^67^ (and the references therein). In particular, we use their software, JuDOKA (https://github.com/KerwannTEP/JuDOKA), to compute diffusion coefficients (*D*_jj_) as a function of eccentricity and semi-major axis for the density profiles in equation (8).

The angular momentum relaxation times can then be estimated using

9 $$\begin{array}{c}{t}_{\mathrm{rx},j(\mathrm{NRR})}=\frac{{j}^{2}}{{D}_{jj,\mathrm{NRR}}},\\ {t}_{\mathrm{rx},j\mathrm{(RR)}}=\frac{{j}^{2}}{{D}_{jj,\mathrm{NRR}}+{D}_{jj,\mathrm{RR}}},\end{array}$$

where *j* is the angular momentum normalized to the circular angular momentum at the same energy. The top and bottom rows corresponds to the time scales without and with resonant relaxation, respectively. We find that both *t*_rx,*j*(NRR)_ and *t*_rx,*j*(RR)_ are about 3 × 10^7^ years for S301, indicating that resonant relaxation is unimportant for this star. This is expected, because of the short Schwarzschild-precession time scale of the star. In other words, the star lies within the Schwarzschild barrier, in which rapid precession suppresses the build-up of coherent torques^68^.

Alternatively, *t*_rx,*j*(NRR)_ ≈ *j*^2^*t*_rx_, which gives results that are consistent with equation (6).

#### Collision time scales

The mean time between collisions for a single target star is

10 $${t}_{{\rm{coll}}}=\frac{1}{n\,\Sigma \,{v}_{{\rm{rel}}}},$$

where *n* is the local number density of potential impactors, *v*_rel_ is the typical relative velocity, and Σ is the collisional cross section. For a star of radius *R*_⋆_ and mass *m*_⋆_ colliding with objects of mass *m*_imp_ and radius *R*_imp_, the cross section including gravitational focusing is

11 $$\Sigma ={\rm{\pi }}{({R}_{\star }+{R}_{\mathrm{imp}})}^{2}\left(1+\frac{{v}_{\mathrm{esc}}^{2}}{{v}_{\mathrm{rel}}^{2}}\right),\qquad {v}_{\mathrm{esc}}^{2}=\frac{2G\,({m}_{\star }+{m}_{\mathrm{imp}})}{({R}_{\star }+{R}_{\mathrm{imp}})}.$$

In the case of S301, *m*_⋆_ ≈ 1.5*M*_⊙_ and *R*_⋆_ ≈ 1.4*R*_⊙_. Then, from equations (8) and (11), the local collision time scale would be about 2.0 × 10^9^ years for stellar-mass black holes and about 2.1 × 10^8^ years for stars.

#### Uncertainties, caveats and other effects

The relaxation and collision time scales we estimate above are local. In principle, the interactions at pericentre can significantly shorten the energy relaxation and collision time scales. A naïve orbit average with the background profile in equation (8) would suggest a reduction at the order of magnitude level. In practice, fewer than one scatterer is expected at the pericentre of S301 for our assumed density profiles, so the local time scales are more realistic.

Our time scale estimates implicitly assume stars are evolving through a localized diffusion process. In reality, angular momentum and energy perturbations from a handful of massive perturbers exhibit a heavy power-law tail, such that orbital evolution would be dominated by large, non-local jumps that can speed up the orbital evolution^69^.

#### Summary of timescales

There is a clear timescale hierarchy at the radius of S301, with *T*_orb_ ≪ *T*_SP_ ≪ *T*_LT_ ≪ *T*_Vec-RLX_ ≪ *T*_coll_, *T*_Sca-RLX_ ≪ *T*_GW_ (Extended Data Table 3), which allows identifying the processes that are relevant for the orbit evolution of S301.

## Online content

Any methods, additional references, Nature Portfolio reporting summaries, source data, extended data, supplementary information, acknowledgements, peer review information; details of author contributions and competing interests; and statements of data and code availability are available at https://doi.org/10.1038/s41586-026-10894-w.

## Supplementary information

Peer Review file

## Figures and Tables

**Extended Data Table 1:**
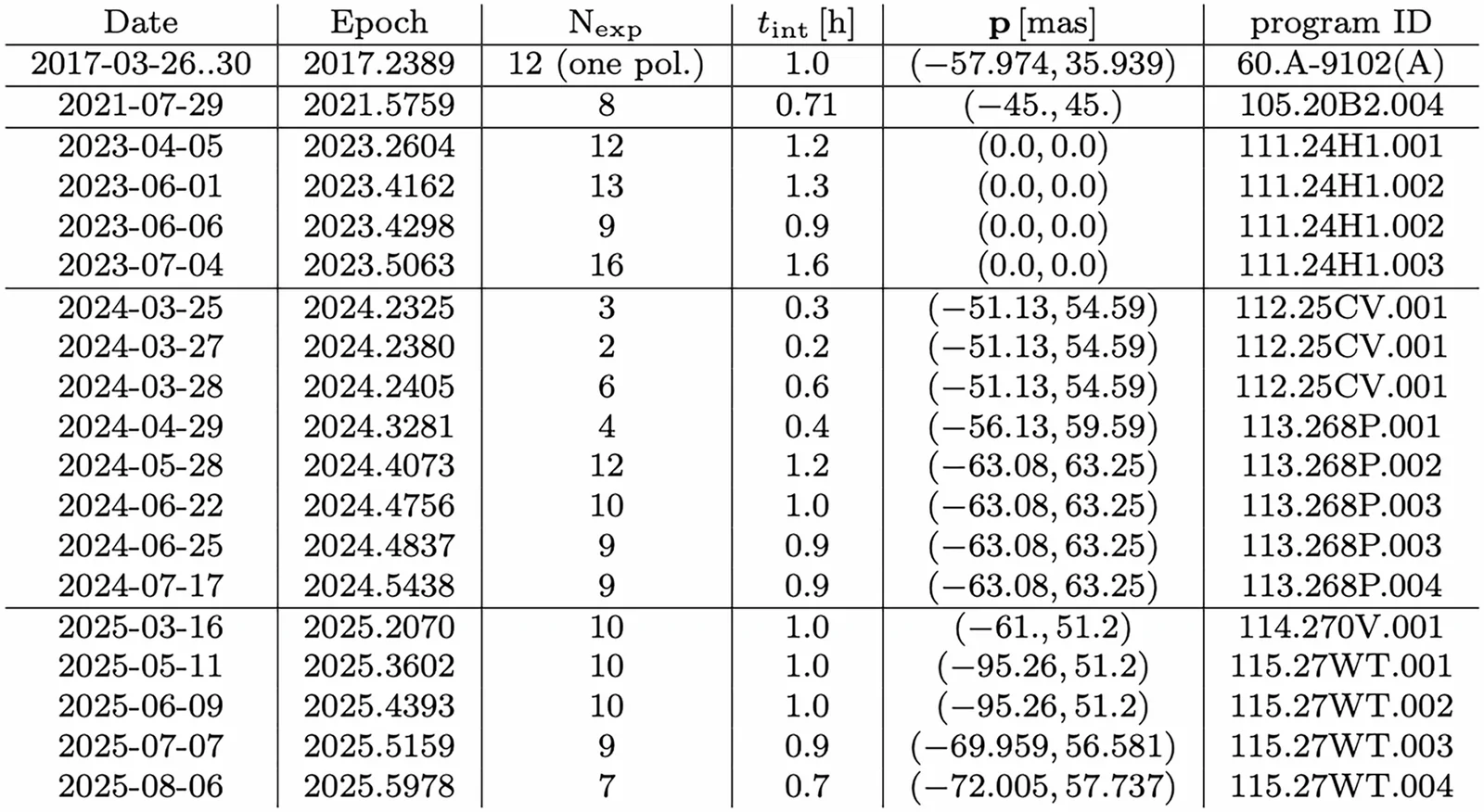
Summary of GRAVITY data that yielded an inference of S301

The date in the first column follows ESO convention and gives the date of the evening of the respective observing night. The epoch is the mean over the time stamps of the ${{\rm{N}}}_{\exp }$ individual GRAVITY exposures. The total accumulated integration time per night is given by *t*_int_, and **p** is the center of the individual pointings with respect to Sgr A*. All data were acquired in low-resolution mode and in two linear polarizations, except for the 2017 dataset, which was partially acquired with both polarizations combined.

**Extended Data Table 2:**
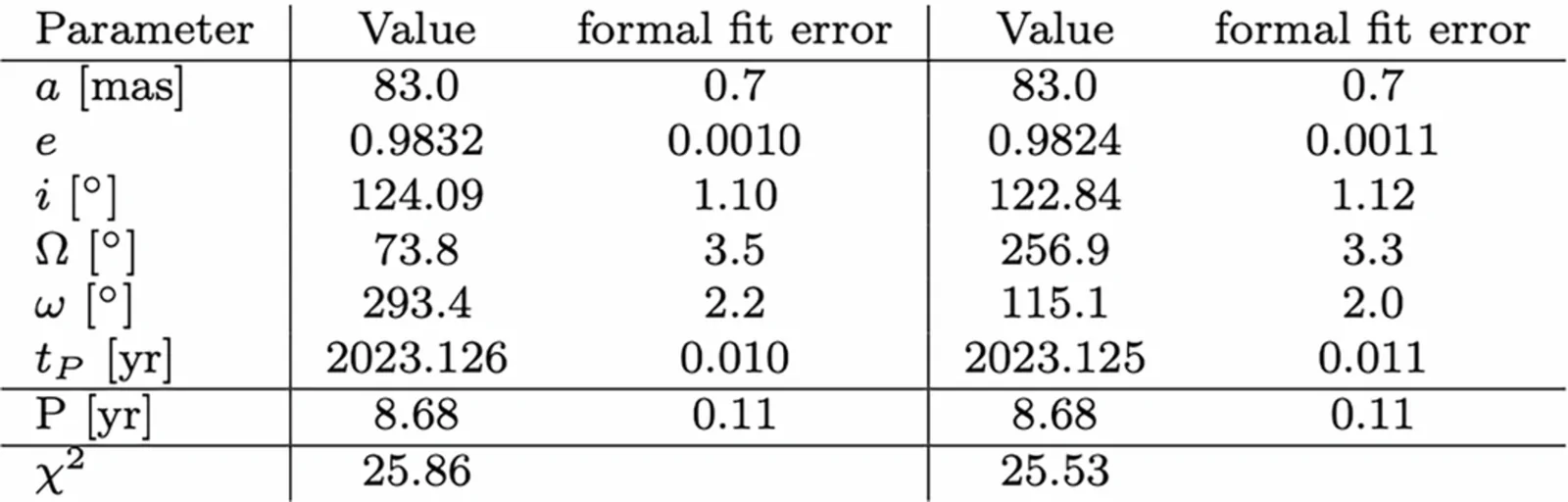
Best-fit orbit parameters for S301

The two sets give the two possible orbit orientations.

**Extended Data Table 3:**
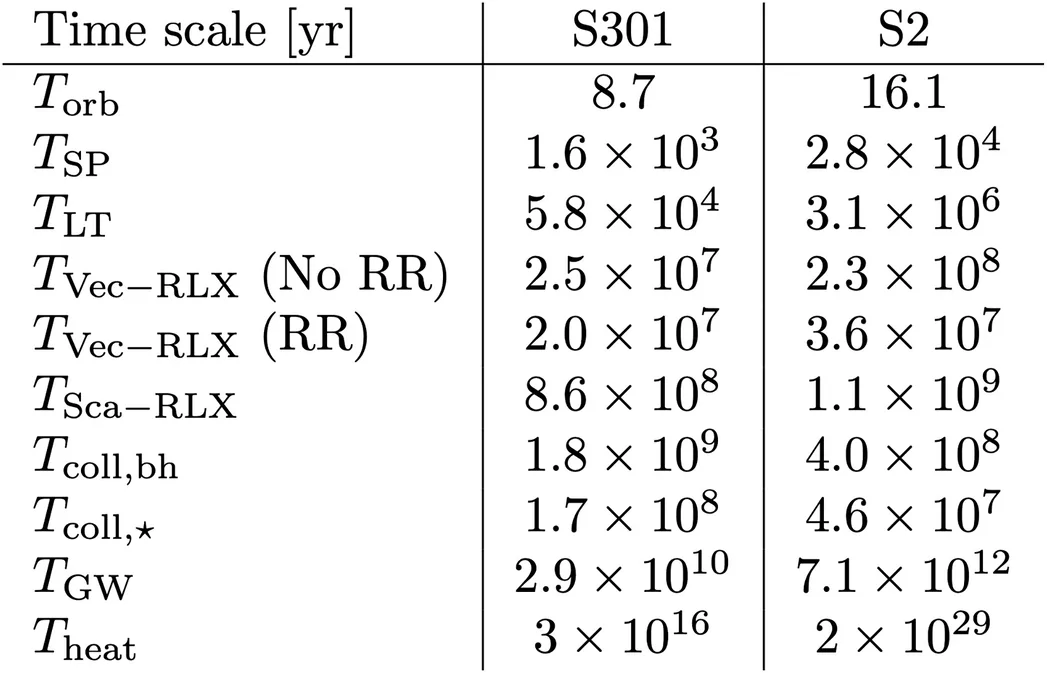
Dynamical time scales for S301 and S2

*T*_orb_ is the orbital period. *T*_SP_ is the Schwarzschild precession time scale, *T*_LT_ for Lense-Thirring precession^72^. *T*_Vec_-RLX refers to the angular momentum relaxation time scale, estimated both with (‘RR’) and without (‘No RR’) resonant relaxation. *T*_Sca_-RLX is the energy relaxation time scale. *T*_coll,bh_ and *T*_coll,⋆_ are the collision time scales with background black holes and stars respectively. The collision and relaxation times are estimated for relaxed stellar density profiles. The gravitational wave inspiral time scale *T*_GW_ is given by^73^, and the energy dissipation time scale *T*_heat_ by^74^ (equation (3)). We assume 1.5*M*_⊙_ and 1.4*R*_⊙_ for the stellar mass and radius of S301, and 15*M*_⊙_ and 4.7*R*_⊙_ for S2. Our estimates of the relaxation times are sensitive to the assumed background density profiles. The relaxation times may be significantly longer if there is a dearth of stellar mass black holes at the radial regime of S301.

## Data Availability

This work is based on observations collected at the European Southern Observatory (ESO) under the ESO programme IDs 60.A-9102(A), 105.20B2.004, 111.24H1.00[123], 112.25CV.001, 113.268P.00[1234], 114.270V.001 and 115.27WT.00[1234]. These data are publicly available through the ESO archive (https://archive.eso.org/cms.html). The astrometric data are available upon request to the corresponding author S.G.

## Code availability

The software to reduce the GRAVITY data is publicly available (https://www.eso.org/sci/software/pipelines/gravity/). The G^R^ source code is available at https://gitlab.mpcdf.mpg.de/gravity/gr_public.git.
