# Supplementary material for: Discovery of a star sensitive to the spin of Sagittarius A*
Source: Nature. 2026 Aug 19;657(8131):359–63. doi: 10.1038/s41586-026-10894-w (PMC13558046; doi:10.1038/s41586-026-10894-w)
Supplement: Supplementary file 1 [file 41586_2026_10894_MOESM1_ESM.pdf]

nature portfolio

Peer Review File

Discovery of a star sensitive to the spin of Sgr A*

Corresponding Author: Dr Stefan Gillessen

Any redactions in this file are there to maintain patient confidentiality, the confidentiality of unpublished data, or to remove third-party material.

This file contains all reviewer reports in order by version, followed by all author rebuttals in order by version.

Version 0:

Reviewer comments:

Referee #1

(Remarks to the Author)
Summary of the key results:

The article reports the discovery and orbit of a star whose pericentre is closer to the central black hole in the Milky Way than any other known star. The relativistic effects on the stellar orbit are strong enough that Lense-Thirring precession should be detectable within a decade, thereby offering the potential to measure the spin of the black hole.

Originality and significance:

The discovery and measurements are made possible by the GRAVITY interferometer, which is a unique facility.

Data & methodology:

The quality of the data is superb and the presentation is careful and clear.

Appropriate use of statistics and treatment of uncertainties:

These are fine.

Conclusions:

The conclusions are robust and reliable.

Suggested improvements:

I have no suggestions for improvements.

References: appropriate credit to previous work?

Yes.

Clarity and context: lucidity of abstract/summary, appropriateness of abstract, introduction and conclusions

All fine. This is an excellent article, well-written and careful. The results are extremely important and I recommend that the article should be published without change and without delay.

Referee #2

(Remarks to the Author)
Summary of Work

===============
In this work, the authors discover a short period star (P~8.7 years), S301, orbiting around our Galaxy's black hole using the GRAVITY interferometer. Its high eccentricity (>0.98) hints at its dynamical history, suggesting that it could be the product of a relatively recent binary capture via the Hill's mechanism. Furthermore, because of its close periapsis (~10x smaller than S0-2's), it is likely sensitive to the spin of SgrA*. With continued observations over the next decade, S301 would provide a direct dynamical measurement of the spin of a black hole, which would be highly impactful and the first of its kind. Nevertheless, there are concerns with the methods, analysis, and data presentation that need to be addressed.

Major Comments
==============
- Why is S300 not shown Figure 1? It should be in the FOV above S63, at least in the 2021 epoch then moving slowly to the right. Is it no longer significantly detected? If so, please explain it, especially given that S301 is a similar magnitude source.

- Please show the 2017 reconstructed image in Figure 1.

- Please put in a table of astrometric points used for fit. This helps the reader to understand the typical astrometric errors of this star (hard to tell from the plots) and will allow for reproducibility.

- Please show the posteriors of the final relativistic orbit fit where the reported statistics are derived from, especially given the result is multimodal.

- Do you have enough kinematic information with just the 2023-2025 data? It would be helpful to quantify "quick and curved motion." Given that this preliminary orbit is used to infer the location of S301 in older data, please show the posteriors along with its associated projected uncertainties with time. How large is the search radius with only the initial points?

- Does the addition of the 2017 and 2021 points change the orbit? If the addition of the points significantly changes the orbit, it could suggest that they are not associated with the 2023-2025 source and/or dominate the final orbit fit.

- How confident is the SX detection? Are S301 and SX of similar magnitude? How do you know that SX is not associated with S301 instead of the other 2021 point? What happens if the SX detection is used for the orbit?

- Why does the flux density of S301 appear to be so different in the 2021 epoch? Does the magnitude of the star change significantly between epochs? A lightcurve or table of magnitude measurements would be helpful, as the figure makes it appear much dimmer.

- What is the confidence of each S301 detection (please quantify "weak" and "strong" inference)? Is S301 found in many more than 5/10 of the GR iterations or just falling at this threshold? Why is this threshold set to 5/10 (seems low)? Are there nearby tentative sources that just fall under this threshold that could be confused for S301? It would be helpful to see a map of nearby sources that don't pass this threshold per epoch. Does this S301 also clearly appear when the data is reduced with other methods (i.e. CLEAN)?

- Does S62 and SgrA* both being within <15 mas in the first 2023 data point cause a bias in the astrometry, especially in the DEC direction? A similar separation was shown to be an issue with S300 in the injection and recovery tests of GRAVITY+2022. Especially given that S62 is around ~10 mas away and slightly brighter, source confusion could be a significant issue. The scatter off the best fit model in the DEC direction tends to be more than RA, but seems worse in 2023 epochs compared to the later years.

- Is the newly presented data mosaiced or are SgrA* and S301 both in the field in the 2025 observations (given they are 70 mas apart and the fiber FWHM is around 65 mas)? If SgrA* is at the fiber edge, does that impact the astrometry of S301 (and/or other stars in the field)?

- Does the confusion event between S62 and SgrA* in 2024/2025 impact the astrometry of S301? Do stars with well-known orbits show a shift in their astrometry in these epochs?

- Errors for S301 seem small given that the average error of a similar magnitude star (S300) in GRAVITY+2022 is around 0.8 mas. S301 appears to have errors as low as 0.25-0.30 mas. Is it because the fiber is centered near the source?

- If errors are underestimated (or there are unaccounted for systematics/biases due to confusion or instrumental effects), a near-edge on (i ~ 90 degrees), but lower eccentricity orbit (<<0.9) could also be possible. At a low eccentricity, it's possible S301 would not be sensitive to SgrA*'s spin (even at a short period). If this is the case, S301 would appear south-east SgrA* in 2021 rather than above in the NW. There appears to be a low probability source in the SE field of GRAVITY+2022. Can you fully rule out the low eccentricity orbit?

- Why is an error of 100 uas assumed as the typical astrometric error in the simulations of the spin sensitivity given that the typical error of this star appears to be 300-1000 uas? At what typical astrometric error would the star not be sensitive to the spin of SgrA*?

Minor Comments

==============
- Figure 1:
-- The magenta is hard to see on black background for S301, choose color with more contrast (such as white).
-- Increase size of axis labels and figure resolution.
-- There is an unlabeled faint detection around 50 mas below SgrA* in 2021. What source is this? How do you know it's not instead associated with S301?

- Figure 2:
-- Put the residuals next to the associated time vs. astrometry plot for easier interpretation.
-- Why do the time vs. RA/DEC plots have error bars on time?
-- Increase size of axis labels and figure resolution.
-- Are orbit error envelopes plotted in these figures? If not, please add them and refer to it in the figure caption.

- Figure 3:
-- In the left figure, why does the time axis go out from 2000 to 2045 while the text says the simulations only go from 2017-2035? Decreasing the time axis extent will also make the plot easier to read.
-- It might be easier to interpret this plot if instead the delta(omega) between the two spin cases is shown on the y-axis, rather than overplotting them.
Increase figure resolution and size of labels.

- Figure 4:
-- Contrast between black and colormap makes lettering hard to see.
-- Increase the size of labels and figure resolution.
-- Labeling the axis will make it easier to interpret.

- Table A.1
-- The 2017 date only has a month, not a day.
-- Why are there 5 2023 astrometry points for S301 but only 4 mentioned in the text and the table?

- Figure B.1
-- Red, green, and orange, brown together may be hard to distinguish.

Referee #3

(Remarks to the Author)
I co-reviewed this manuscript with one of the reviewers who provided the listed reports.

Referee #4

(Remarks to the Author)
I read with interest the paper "Discovery of a start sensitive to the spin of Sgr A*" by Abd El Dayem et al. I find this to be a very exciting development in the field with potential significant implications. I have a number of suggestions/comments for the author, which I detail below, before I can recommend the paper for publication.

- My biggest surprise is related to the clear detection of the Schwarzschild precession of the orbit, driven by the fact that the 2017 and the 2025 observations correspond to the same orbital phase (near the apoapsis) but appear at different declinations in the sky (Fig 2). Visually, the error bars appear to be significantly smaller than the size of the precession. Combined with the fact that the periapsis of this star is an order of magnitude smaller than the periapsis of S2, I would expect that this orbit would provide the strongest test of PPN parameters to date around a black hole.

Just looking at the data, I expect the limits on the 1st order PPN parameters to be significantly better than the equivalent limit from the EHT or from LIGO/Virgo tests (albeit for larger distances from the black hole) and might even be better than the limit from the double pulsar at similar distances from the compact object (albeit for a black hole and not for a neutron star). See discussion around Section 6.2 and Fig 26 of
Event Horizon Telescope Collaboration, Akiyama, K., Alberdi, A., et al.\ 2022, \apjl, 930, 2, L17. doi:10.3847/2041-8213/ac6756
I find this to be very significant.

It is also interesting that, because of the large eccentricity of the orbit, the 2nd order PPN effect becomes comparable to the Lense-Thirring effect (due to spin) when the spin of the black hole is <0.1/|cos beta| (if I did the math right). For reasonable values of beta and spin, the 2nd order PPN effects might become measurable before spin effects and this would also be a very important result. See Will, C.~M.\ 2018, \prl, 120, 19, 191101. doi:10.1103/PhysRevLett.120.191101

I would strongly encourage the authors to explore these possibilities in their paper (at least at an order-of-magnitude level), which will give the opportunity for this paper to report new physics results as opposed to just promises for future observations.

- In section 4.2, I find the use of the word "randomly" for the chosen orientation of the spin to be misleading: this word implies that the authors are following a conservative approach when, in reality, this is by far the most optimistic configuration. I would

make the left panel of Figure 3 with the same values of beta and lambda as in the right panel and then, perhaps, plot the difference between the x=0 and x=1 case in order to illustrate better the effect. In practice, what one measures is x*cos(beta) and, perhaps, the authors can discuss their results in terms of that quantity.

Incidentally, the uncertainty in spin from the mock data is quoted as 0.2 in the figure caption but as 0.1 in the text.

* Minor

- Contrary to the first sentence of the paper, a black hole has three degrees of freedom, if one includes charge. Perhaps the authors can say "An astrophysical black hole in GR..."

- It will be appropriate for the authors to acknowledge the work of the Keck/UCLA group in measuring the properties of Sgr A* via monitoring of stellar orbits, together with reference [8].

- Similarly, around the discussion of Fig 3, it would be appropriate to acknowledge early work that quantified possible constraints on the black-hole spin through monitoring of stars closer than S2, e.g.,
Weinberg, N.~N., Milosavljevi{\'c}, M., \& Ghez, A.~M.\ 2005, \apj, 622, 2, 878. doi:10.1086/428079
Ang{\'e}lil, R., Saha, P., \& Merritt, D.\ 2010, \apj, 720, 2, 1303. doi:10.1088/0004-637X/720/2/1303
Psaltis, D., Wex, N., \& Kramer, M.\ 2016, \apj, 818, 2, 121. doi:10.3847/0004-637X/818/2/121

Version 1:

Reviewer comments:

Referee #2

(Remarks to the Author)
Summary of Work
===============
In this work, the authors discover a short period star (P~8.7 years), S301, orbiting around our Galaxy's black hole using the GRAVITY interferometer. Because of its close periapsis (~10x smaller than S0-2's), it is likely sensitive to the spin of SgrA*. With continued observations over the next decade, S301 would provide a direct dynamical measurement of the spin of a black hole, which would be highly impactful and the first of its kind. The only remaining concern is in the data availability.

Major Comments
==============
The astrometric points for this star should be published in a table somewhere in this paper given it is a key result and primary data product used for all conclusions of this study. The reduction of the GRAVITY data with the gravity resolve software is non-trivial and also requires knowledge of the orbits of stars and their respective fluxes (none of which are clearly referenced). In this case, It would be difficult for someone to reproduce the points and the astrometric data product. The publication of the points is necessary for accessibility and for others to build upon this work.

Referee #3

(Remarks to the Author)
I co-reviewed this manuscript with one of the reviewers who provided the listed reports.

Referee #4

(Remarks to the Author)
I would like to thank the authors for carefully addressing my comments. I find the revised version to be suitable for publication.

Open Access This Peer Review File is licensed under a Creative Commons Attribution 4.0 International License, which permits use, sharing, adaptation, distribution and reproduction in any medium or format, as long as you give appropriate credit to the original author(s) and the source, provide a link to the Creative Commons license, and indicate if changes were made.
In cases where reviewers are anonymous, credit should be given to 'Anonymous Referee' and the source.
The images or other third party material in this Peer Review File are included in the article's Creative Commons license, unless indicated otherwise in a credit line to the material. If material is not included in the article's Creative Commons license and your intended use is not permitted by statutory regulation or exceeds the permitted use, you will need to obtain permission directly from the copyright holder.
To view a copy of this license, visit https://creativecommons.org/licenses/by/4.0/

Answers

Following the editorial guide lines, we have overall shortened the text, and also have moved major parts of the discussion section to the appendix. These edits have not changed content. The edits that follow the referees' suggestions, on the other hand, yielded very valuable additions, and we have indicated those changes in blue font.

Referee 1

We thank you for your positive review!

Referee 2 + 3

We truly thank you for your thorough review of the manuscript and the detailed comments. We have tried to address all of them, and hope that this improved the manuscript significantly. Let us go through the comments one-by-one.

Major Comments
==============

- Why is S300 not shown Figure 1? It should be in the FOV above S63, at least in the 2021 epoch then moving slowly to the right. Is it no longer significantly detected? If so, please explain it, especially given that S301 is a similar magnitude source.

The pointing position in 2021 was at RA/Dec = -45/+45 mas, i.e. NW of SgrA*, centered roughly on where S2 was at the time. S300 was at around -35/-18 mas. i.e. 64 mas away. At this distance, S300 is damped by the Gaussian acceptance of GRAVITY fibers by several magnitudes, putting it beyond the limiting magnitude. In the later years, it is truly outside of the field of view. The GRAVITY+2022 paper used the fiber pointing on Sgr A*. We have not changed the text for this point.

- Please show the 2017 reconstructed image in Figure 1.

We show the 2017 image in the appendix now, Figure B.2. The shorter exposure time and overall lower data quality, plus the vicinity to the bright star S2 make the detection more challenging.

- Please put in a table of astrometric points used for fit. This helps the reader to understand the typical astrometric errors of this star (hard to tell from the plots) and will allow for reproducibility.

We have improved Figure 2, such that one can see more details now. We note that one error bar (in one coordinate) was wrong in our original data table, which we have fixed now.

As stated in the data availability statement, the raw data are publicly available through the ESO archive, including the software to reduce the GRAVITY data.

- Please show the posteriors of the final relativistic orbit fit where the reported statistics are derived from, especially given the result is multimodal.

We show a posterior in the appendix now (Figure C3). Note, that the fit parameters are well constrained and not multimodal. There is an ambiguity of the tilt of the orbit due to the absence of radial velocity information. Beyond that, the fit is very well behaved.

- Do you have enough kinematic information with just the 2023-2025 data? It would be helpful to quantify "quick and curved motion."

Yes, one gets an orbit also with the 2023-2025 data only. It has a larger uncertainty obviously, and in particular because the pericenter passage is outside of the data range then. The star moved in 2023 with a velocity of around (-44 / +27) mas/yr (in RA / Dec), and an acceleration significant at the +3/+5 sigma level. We have indicated this in the text,

- Given that this preliminary orbit is used to infer the location of S301 in older data, please show the posteriors along with its associated projected uncertainties with time. How large is the search radius with only the initial points?

We show the back-prediction now in figure A1. The star is reasonably close to where we expect it.

- Does the addition of the 2017 and 2021 points change the orbit? If the addition of the points significantly changes the orbit, it could suggest that they are not associated with the 2023-2025 source and/or dominate the final orbit fit.

Figure A1 should answer this point as well. While it is clear that the orbit changes somewhat due to the addition of pre-pericenter points, that have a long temporal lever arm, the figure illustrates that there is no doubt for the identification of the star.

An additional point worth mentioning is that by now, we have obtained a first 2026 data point for S301, and it is precisely where the orbit had it predicted.

- How confident is the SX detection? Are S301 and SX of similar magnitude? How do you know that SX is not associated with S301 instead of the other 2021 point? What happens if the SX detection is used for the orbit?

We could not re-identify this detection in another period, therefore have not yet assigned a star number. The position of SX does not match the orbit, see figure A1.

- Why does the flux density of S301 appear to be so different in the 2021 epoch? Does the magnitude of the star change significantly between epochs? A lightcurve or table of magnitude measurements would be helpful, as the figure makes it appear much dimmer.

We have added a light curve to Figure 1. The light curve is not unusual for a faint source, and consistent with constant brightness.

- What is the confidence of each S301 detection (please quantify “weak” and “strong” inference)? Is S301 found in many more than 5/10 of the GR iterations or just falling at this threshold? Why is this threshold set to 5/10 (seems low)? Are there nearby tentative sources that just fall under this threshold that could be confused for S301? It would be helpful to see a map of nearby sources that don’t pass this threshold per epoch.

In the post-pericenter data, S301 is detected in all iterations, i.e. there is no doubt on the inference, and we call it “strong”. A weak inference is used for an inference in around 50% of the samples.

Further, we note that the fitting algorithms used, and the CLEAN images confirm a source in the positions derived from the GR imaging code.

The detection limit is considerably deeper than S301’s magnitude, probably around mK=20 (determined via injection tests and currently being put together in a separate publication). This is for example why the “unlabeled faint detection around 50 mas below SgrA* in 2021“ does not carry a label. At that level, detections might be spurious.

Does this S301 also clearly appear when the data is reduced with other methods (i.e. CLEAN)?

Yes, we have also employed CLEAN techniques; and we can recover S301, at least for the post-pericenter data. We show now an example in figure B4.

- Does S62 and SgrA* both being within <15 mas in the first 2023 data point cause a bias in the astrometry, especially in the DEC direction? A similar separation was shown to be an issue with S300 in the injection and recovery tests of GRAVITY+2022. Especially given that S62 is around ~10 mas away and slightly brighter, source confusion could be a significant issue. The scatter off the best fit model in the DEC direction tends to be more than RA, but seems worse in 2023 epochs compared to the later years.

Note: S62 and SgrA* are only close to being confused. The point is well taken, but it does not apply actually: For the known sources, the G^R code does the same as a model fit: The sources are fit in position and flux. And as in all our fitting codes, such separations are well handled in fitting. If one would need to infer a previously unknown source, however, the imaging part of the code is what matters, and here one could ask whether one introduces biases. The scatter in the corrected data set is now also more regular (*).

- Is the newly presented data mosaiced or are SgrA* and S301 both in the field in the 2025 observations (given they are 70 mas apart and the fiber FWHM is around 65 mas)? If SgrA* is at the fiber edge, does that impact the astrometry of S301 (and/or other stars in the field)?

Indeed, in 2025 the sources SgrA* and S301 are too far apart for observing in single mode. We hence point the fiber part of the time to SgrA*, and part of the time to S301 (where also S21 is in the field). The two fields are linked to each other astrometrically via the metrology system of GRAVITY.

- Does the confusion event between S62 and SgrA* in 2024/2025 impact the astrometry of S301? Do stars with well-known orbits show a shift in their astrometry in these epochs?

We have not seen this effect, and we can in all epochs actually model both S62 and SgrA*, and break the confusion. So by modeling both sources, we do not introduce a bias. This might be a misunderstanding due to the caption of figure 1, which we have updated now.

- Errors for S301 seem small given that the average error of a similar magnitude star (S300) in GRAVITY+2022 is around 0.8 mas. S301 appears to have errors as low as 0.25-0.30 mas. Is it because the fiber is centered near the source?

The error bars in GRAVITY+2022 were simply the size of the pixels of the grid. Indeed, the uncertainties are typically in the range below 0.3mas.

- If errors are underestimated (or there are unaccounted for systematics/biases due to confusion or instrumental effects), a near-edge on (i ~ 90 degrees), but lower eccentricity orbit (<<0.9) could also be possible. At a low eccentricity, it's possible S301 would not be sensitive to SgrA*'s spin (even at a short period). If this is the case, S301 would appear south-east SgrA* in 2021 rather than above in the NW. There appears to be a low probability source in the SE field of GRAVITY+2022. Can you fully rule out the low eccentricity orbit?

Yes, there is only one solution to the orbit fit. Note that the appearance of the ellipse on sky is not what is fit. We fit RA and Dec as a function of time; we have updated the text in section 2 for making this clearer. The chi^2 value we get does not point to underestimated error bars.

- Why is an error of 100 uas assumed as the typical astrometric error in the simulations of the spin sensitivity given that the typical error of this star appears to be 300-1000 uas? At what typical astrometric error would the star not be sensitive to the spin of SgrA*?

The current data are not yet representative for the final, expected performance of GRAVITY+, for which we think uncertainties of around 100µas are realistic. The key difference will be the laser-guide-star assisted adaptive optics, which will lead to a much higher and much more stable flux injection into the fibers of the beam combiner, and the upcoming upgrade of all instrument optics with factor 10 improved surface figure. Further, the newly installed adaptive optics system was only available since (beginning of) 2025. The effect of having more light in the fiber is non-linear, as the fringe-tracking then performs better, one gets few drop-outs, etc. Overall, the exact number used for the simulation does not matter very much, and the round value chosen is not an overly optimistic number.

Minor Comments
==============
- Figure 1:
-- The magenta is hard to see on black background for S301, choose color with more contrast (such as white).
-- Increase size of axis labels and figure resolution.
-- There is an unlabeled faint detection around 50 mas below SgrA* in 2021. What source is this? How do you know it's not instead associated with S301?

- We have improved the figure.
- This faint peak cannot be associated with any star, it is most likely noise. There is no known orbit that fits this point.

- Figure 2:
-- Put the residuals next to the associated time vs. astrometry plot for easier interpretation.
-- Why do the time vs. RA/DEC plots have error bars on time?
-- Increase size of axis labels and figure resolution.
-- Are orbit error envelopes plotted in these figures? If not, please add them and refer to it in the figure caption.

- We have improved the figure.

- Figure 3:
-- In the left figure, why does the time axis go out from 2000 to 2045 while the text says the simulations only go from 2017-2035? Decreasing the time axis extent will also make the plot easier to read.

The left figure serves to illustrate the order of magnitude of the relativistic effects. It does not involve any simulated data. The right figure shows a simulation in the sense of creating data points and fitting them. We have chosen 2035 to illustrate that within a decade a spin measurement seems in reach.

-- It might be easier to interpret this plot if instead the delta(omega) between the two spin cases is shown on the y-axis, rather than overplotting them.
-- Increase figure resolution and size of labels.

We have included also delta(omega) in the plot now. The original plot is kept as it useful as it illustrates the size of the LT effect compared to Schwarzschild, and nicely shows that the LT precession of S301 is comparable to the Schwarzschild precession of S2.

- Figure 4:
-- Contrast between black and colormap makes lettering hard to see.
-- Increase the size of labels and figure resolution.
-- Labeling the axis will make it easier to interpret.

We have improved the figure. It is now Fig. D7.

- Table A.1
-- The 2017 date only has a month, not a day.

We have added the days, as this point combines data from a few nights.

-- Why are there 5 2023 astrometry points for S301 but only 4 mentioned in the text and the table?

This is a mistake. There are five 2023 data points.

- Figure B.1
-- Red, green, and orange, brown together may be hard to distinguish.

We have improved the figure. It is now figure B2.

Referee 4

We thank you very much for your suggestions, which have sparked several interesting discussions. Basically, there are two major points:

1)
Regarding the respective importance of the 2PN (monopolar) and 1.5PN (leading-order spin) effects on the in-plane precession of the pericenter, we have carefully checked the formulas and find the following. We use the in-plane pericenter shift formulas as provided by Abd El Dayem et al. (2026, arxiv 2505.24789), Eqs. 38-39. The ratio r of the 2PN to 1.5PN shift reads :

r = 3/16 * epsilon^{1/2} * (2+e^2)/(cos(beta) * chi)

using the paper's notation, and with epsilon = GM/(p c^2) being the PN small parameter.

We have plotted the evolution of this ratio with cos(beta)*chi in the enclosed figure. This shows the standard effect that the 2PN term dominates the 1.5PN term as the spin decreases, but it also shows that the impact of the eccentricity is rather limited. It does change the spin value at which the 2PN term starts to dominate, but only marginally, by around 50%. Our conclusion is that this eccentricity effect is too tiny to justify a dedicated discussion in the paper, but we added a sentence to note the importance of the 2PN terms:

"*Future robust spin constraints will require modeling up to 2PN order to avoid systematic biases at low spins.*"

(The corresponding paragraph of the text has now moved to the appendix.)

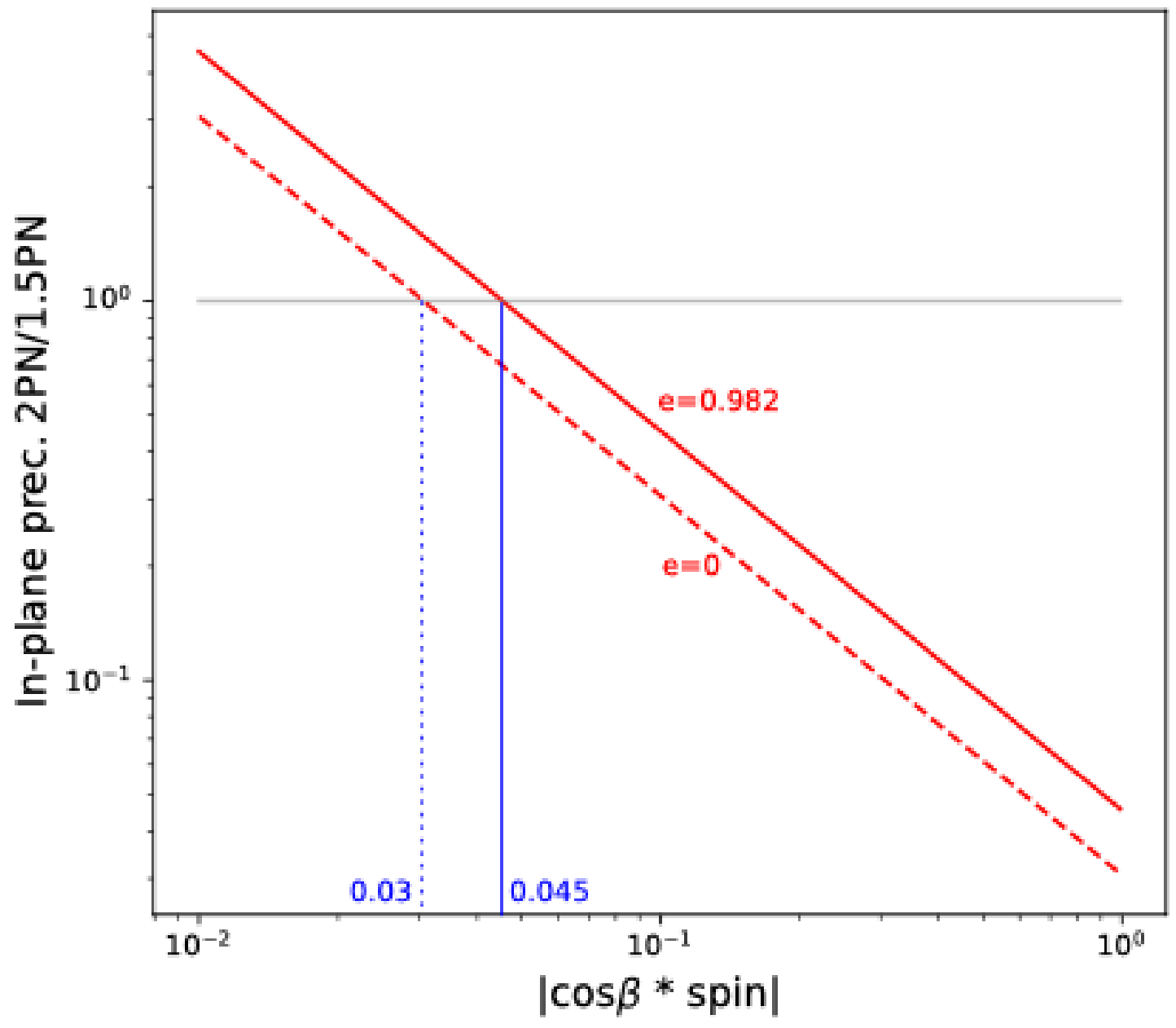

2)
Regarding the constraints one might be able to derive from the clearly visible Schwarzschild precession:

This is a very valid point, and we have looked into it following the referee's suggestion. Unfortunately, the uncertainties of the first two data points are too large to make any firm statement; and the S2-derived constraint remains (for the moment) the stronger one. We have added the following to the discussion section:

„ S301's Schwarzschild precession, about ten times larger than that of S2, offers the opportunity to tighten constraints on possible deviations from general relativity (e.g., strong-field PPN parameters) and on the extended mass distribution between the apocenters of S2 and S301. The current dataset is consistent with general relativity at the 1PN level, although the resulting PPN bounds are weaker than those derived from S2 alone [14, 34] with fSP = 0.94 ± 0.88. A joint analysis may nev- ertheless improve these constraints, and continued long-term monitoring of S301will significantly strengthen them.“

For the minor points:

- We have omitted the word "randomly" in former section 4.2 (now Appendix D). Looking at figure D7 (former figure 4), we don't think this the most optimistic case. (But the choice of chi=1 is of course.)

- We have made consistent the statement on how precise the spin amplitude chi can be determined ("<0.2").

- The first sentences read: "Black holes in general relativity have just two additional degrees of freedom beyond mass: spin and charge. Astrophysically relevant is the spin." So this should be fine.

- We have added the 2008 + 2009 papers from Ghez & Gillessen in the introduction for acknowledging the work that led to this discovery. Further, we added the 2012 Do et al. Science paper on the detection the redshift.

- Thanks for pointing out the three additional references. This was obviously an oversight, and given the relevance of these earlier papers, we have added references to them to the introduction section.
